# A Novel Human Pluripotent Stem Cell-Derived Neural Crest Model of Treacher Collins Syndrome Shows Defects in Cell Death and Migration

**DOI:** 10.1089/scd.2017.0234

**Published:** 2019-01-10

**Authors:** Felipe Serrano, William George Bernard, Alessandra Granata, Dharini Iyer, Ben Steventon, Matthew Kim, Ludovic Vallier, Laure Gambardella, Sanjay Sinha

**Affiliations:** ^1^ Anne McLaren Laboratory, Wellcome Trust-Medical Research Council Cambridge Stem Cell Institute, Department of Medicine, University of Cambridge, Cambridge, United Kingdom.; ^2^ Division of Clinical Neurosciences, Clifford Allbutt Building, Cambridge Biomedical Campus, Cambridge, United Kingdom.; ^3^ Department of Genetics, University of Cambridge, Cambridge, United Kingdom.

**Keywords:** neural crest, differentiation, disease modeling, Treacher Collins syndrome, human pluripotent stem cells

## Abstract

The neural crest (NC) is a transient multipotent cell population present during embryonic development. The NC can give rise to multiple cell types and is involved in a number of different diseases. Therefore, the development of new strategies to model NC in vitro enables investigations into the mechanisms involved in NC development and disease. In this study, we report a simple and efficient protocol to differentiate human pluripotent stem cells (HPSC) into NC using a chemically defined media, with basic fibroblast growth factor 2 (FGF2) and the transforming growth factor-β inhibitor SB-431542. The cell population generated expresses a range of NC markers, including P75, TWIST1, SOX10, and TFAP2A. NC purification was achieved in vitro through serial passaging of the population, recreating the developmental stages of NC differentiation. The generated NC cells are highly proliferative, capable of differentiating to their derivatives in vitro and engraft in vivo to NC specific locations. In addition, these cells could be frozen for storage and thawed with no loss of NC properties, nor the ability to generate cellular derivatives. We assessed the potential of the derived NC population to model the neurocristopathy, Treacher Collins Syndrome (TCS), using small interfering RNA (siRNA) knockdown of TCOF1 and by creating different TCOF1^+/−^ HPSC lines through CRISPR/Cas9 technology. The NC cells derived from TCOF1^+/−^ HPSC recapitulate the phenotype of the reported TCS murine model. We also report for the first time an impairment of migration in TCOF1^+/−^ NC and mesenchymal stem cells. In conclusion, the developed protocol permits the generation of the large number of NC cells required for developmental studies, disease modeling, and for drug discovery platforms in vitro.

## Introduction

In mammalian development, neural crest (NC) cells are a transient multipotent population arising from the neural plate border, usually contemporaneously with neural tube closure [1]. These migratory cells invade adjacent tissues and differentiate into multiple cell types, including mesenchymal stem cells (MSC), vascular smooth muscle cells (SMC), adipocytes, osteocytes, chondrocytes, melanocytes, glia, and Schwann cells [2]. NC cells are also implicated in a broad range of pathologies making the in vitro generation of these cells of high clinical relevance.

The development and function of the NC have been well characterized in animal models, including the chick, zebrafish, and mouse [3–6]. NC specification and induction depend on signaling molecules and transcription factors, whose actions are coordinated during gastrulation and neurulation. The neural plate border is specified by the cross talk between BMP, WNT, and fibroblast growth factor (FGF) signaling [7]. Initially, intermediate levels of BMP signaling induce neural folds with anterior character over the whole neural plate border. Next, the posterior regions of the neural plate border are transformed into NC under the effects of FGF, WNT, and retinoic acid [8].

After specification, NC cells undergo epithelial–mesenchymal transition (EMT) before delaminating from the border of the neuroepithelium [9]. Subsequently the arising NC cells migrate in response to specific cues and express specific proteins, including Twist [10], Sox10 [3,11], and p75 [12]. These factors control cellular events such as delamination, cell proliferation, migration, and differentiation [13–15]. Defects in the complex processes that choreograph NC development are involved in congenital human diseases known as neurocristopathies.

Human embryonic stem cells (HESC) and human induced pluripotent stem cells (HIPSC) are collectively referred to as human pluripotent stem cells (HPSC). Some of the most relevant NC differentiation protocols from HPSC are based on initially producing in vitro neuroectoderm [16–18] and then purifying the nascent NC population using different approaches such as dual SMAD inhibition [18–21] and/or WNT pathway activation [22–24]. A common limitation of these approaches is the use of undefined basement membrane extracts as a coating for cell adhesion, while only a few protocols have so far been validated in vivo [21,24–26]. Further optimization and validation of NC in vitro differentiation protocols would be valuable for studies into human development and disease.

Treacher Collins Syndrome (TCS) [27,28] (OMIM: 154500) is a neurocristopathy resulting in a severe congenital craniofacial disorder, which occurs one in every 50,000 births [29]. TCS is primarily associated with autosomal dominant haploinsufficiency-inducing mutations in the *TCOF1* gene located on chromosome 5 [30], which lead to deficient ribosome biogenesis [31]. *Tcof1* is expressed broadly throughout the mouse embryo, with high activity in the neuroepithelium where it plays an essential role in cell survival [32]. Extensive apoptosis of the neuroepithelial progenitor has been reported in TCS, resulting in impaired NC differentiation and subsequent defects in craniofacial development [32].

In this study, we report a complete differentiation protocol using simple conditions that permits the generation of the NC from HPSC using a combination of FGF signaling and TGF-β inhibition. Derived NC cells are proliferative, can be maintained over multiple passages, can differentiate to a variety of cell types in vitro, and have been validated in a developmental chick embryo model. Furthermore, we have utilized CRISPR/Cas9 technology to generate *TCOF1^+/^*^−^ lines as a model of TCS and have revealed abnormalities in cell migration, which may play a role in the underlying pathology of the disease. The development of this protocol permits the generation of NC and its derivatives in a chemically defined media (CDM) for developmental studies, disease modeling, and drug discovery.

## Materials and Methods

### HPSC culture

HESCs (H9s line; WiCell, Madison, WI) and HIPSC (BBHX8 line [33], Cambridge Biomedical Research Centre HIPSC core facility) called together as HPSC were maintained with CDM plus bovine serum albumin fraction A (CDM-BSA), as previously described [34]. CDM-BSA was composed of Iscove's modified Dulbecco's medium (Thermo Fisher Scientific) plus Ham's F12 NUT-MIX (Thermo Fisher Scientific) medium in a 1:1 ratio, supplemented with GlutaMAX-I (Thermo Fisher Scientific), BSA (5 mg/mL; Europa Bioproducts), chemically defined lipid concentrate (Thermo Fisher Scientific), transferrin (15 μg/mL; Roche Diagnostics), insulin (7 μg/mL; Roche Diagnostics), and monothioglycerol (450 μM; Sigma). For the maintenance of HPSC, CDM-BSA was supplemented with Activin A (10 ng/mL; R&D Systems) and FGF2 (12 ng/mL; R&D Systems), and cells were maintained on tissue culture treated plastic coated with 0.1% gelatin (Sigma-Aldrich).

HPSC were also cultured and maintained in TeSR^™^-E8^™^ media (STEMCELL Technologies) using Vitronectin XF (STEMCELL Technologies) as chemically defined xeno-free cell culture matrix.

### HPSC differentiation to NC

For NC differentiation, HPSC were detached from gelatin-coated plates using 1 mg/mL collagenase IV (Gibco). Clumps were triturated, counted, and plated at a density of 300 colonies/well in 0.1% gelatin-coated six well plates in CDM-BSA supplemented with Activin A (10 ng/mL; R&D Systems) and FGF2 (12 ng/mL; R&D Systems), referred to herein as CDM-BSA+FA.

After 24 h in CDM-BSA, the media was changed to CDM-polyvinyl alcohol (PVA) supplemented with FGF2 (12 ng/mL; R&D Systems) and SB-431542 (10 μM; Tocris), referred to herein as FSB, for 4 days without splitting. CDM-PVA has the same composition as CDM-BSA, with PVA (1 mg/mL; Sigma) instead of BSA. On day 4 in FSB, the differentiating HPSC was dissociated using TrypLE Express^™^ (Gibco) and seeded as single cells at a 1:3 ratio on 0.1% gelatin-coated plates in FSB. The single cells were maintained in FSB (with daily media changes) on 0.1% gelatin-coated plates over several passages; the splitting of those single cells was performed every 3–4 days (80%–90% confluence) at 1:3 ratio using TrypLE Express (Gibco).

For differentiating HPSC grown on Vitronectin XF and maintained with TeSR-E8, the HPSC colonies were detached using 0.5 mM EDTA (Gibco). Clumps were counted and seeded at a density of 300 colonies/well in vitronectin XF-coated six well plates in TeSR-E8 media. After 24 h in TeSR-E8, the media was changed to FSB, and cells were maintained in FSB on vitronectin XF without splitting for 4 days. At day 4 in FSB, the differentiating HPSC were dissociated with TrypLE Express, seeded as single cells on 0.1% gelatin-coated plates and split every 3–4 days as described above.

### Quantitative real-time polymerase chain reaction

Total RNA was extracted using the RNeasy Mini Kit (Qiagen). Complementary DNA (cDNA) was synthesized from 250 ng total RNA using the Maxima First Strand cDNA Synthesis Kit (Thermo Fisher Scientific). Quantitative real-time polymerase chain reaction (qRT-PCR) mixtures were prepared with the FAST-SYBR Green Master Mix (Thermo Fisher Scientific) and analyzed on a 7500 Fast Real-time PCR system (Thermo Fisher Scientific). C_T_ values were normalized to porphobilinogen deaminase (PBGD). Primer sequences are listed in Supplementary Table S1 (Supplementary Data are available online at www.liebertpub.com/scd).

### Flow cytometry

Cells were fixed with Cytofix/Cytoperm Fixation solution (BD Biosciences) for 20 min at 4°C, then washed with Perm Wash Buffer/phosphate-buffered saline (PBS, 1 × ; BD Biosciences) and permeabilized with Perm Wash Buffer/PBS +0.1% Triton X-100 for 30 min. Cells were blocked with 3% BSA in 1 × Perm Wash Buffer at room temp for 30 min. After blocking, cells were incubated in primary antibody (Supplementary Table S2) diluted in 1 × Perm Wash Buffer +0.1% Triton X-100 4°C for 45 min. Alexa Fluor^®^-tagged secondary antibody was added after primary incubation for 1 h at room temperature. Samples were run on a Beckman Coulter CyAn-ADP flow cytometer, and subsequent datasets were analyzed using FlowJo software.

### Immunocytochemistry

Adherent cells were fixed using 4% PFA, permeabilized with 0.5% Triton X-100 in PBS (Sigma), and blocked with PBS +3% BSA for 60 min at room temperature. Primary antibody (Supplementary Table S2) incubations were performed at 4°C overnight and Alexa Fluor tagged secondary antibodies applied for 45 min at room temperature the following day. Nuclei were counterstained with DAPI (0.1 μg/mL; Sigma). Images were acquired on a Zeiss LSM 700 confocal microscope and analyzed with ImageJ software.

### Western blotting

Cells were lysed in RIPA buffer containing phosphatase inhibitor cocktail (Sigma) and protease inhibitor cocktail (Sigma) on ice for 15 min, and protein content was quantified using a Pierce BCA Protein Assay Kit (Thermo Fisher Scientific). Ten micrograms of protein per sample was resolved by electrophoresis and transferred to PVDF membranes. Membranes were blocked for 1 h at room temperature with 5% milk in Tris-Buffered Saline containing 0.1% Tween-20 (TBS-t; Sigma) and incubated overnight with either anti-TCOF1 (1:1,000; Abnova) or anti-beta actin (1:10,000; Sigma). Membranes were washed with TBS-t and incubated with horseradish peroxidase (HRP)-conjugated secondary antibodies for 1 h at room temperature. Membranes were washed and developed using the Pierce ECL2 western blotting substrate (Thermo Fisher Scientific).

### Migration assays

NC and MSC were plated onto six-well plates and allowed to form a confluent monolayer. The cell monolayer was then scratched in a straight line to make a “scratch wound” with a 1-mL pipette tip, and the cell debris was removed by washing the cells with phosphate-buffered saline (PBS). Cells were maintained in FSB (for NC cells) or Dulbecco's modified Eagle's medium (DMEM) +10% FBS (for MSC), and images of the closure of the scratch were captured at different time points as indicated.

A chemotaxis assay was performed using the CytoSelect^™^ 24-Well Cell Migration and Invasion Assay Combo Kit (Cell Biolabs) following the manufacturer's instructions. Briefly, 5 × 10^5^ cells were plated in a prewarmed 24-well migration plate in FSB media. The chemoattractant FGF8B (30 ng/mL; R&D Biosystems) was added separately to FSB media. Cell media was added to the lower well of the migration plate. FSB media was used as a control. Cells were incubated for 8 h at 37°C, and quantification was performed following the manufacturer's instructions.

For single cell analysis of cell migration, cells were imaged real time on an IN Cell Analyzer 2200 (GE Healthcare Life Sciences) with images collected every 30 min for a 12-h period. Cells were tracked using the Pointing Cell Tracking plugin for ImageJ (https://imagej.nih.gov/ij/plugins/pointing-cell-tracking/index.html), and migratory profiles were generated using the freely available Chemotaxis and Migration Tool from Ibidi (ibidi.com/chemotaxis-analysis/171-chemotaxis-and-migration-tool.html).

### Microarray hybridization and analysis

RNAs isolated from H9s HESC, neuroectoderm [34], and NC passage 2 and 7 (NC P2, NC P7) were hybridized with Illumina Human HT-12 BeadChip (Illumina, Inc., San Diego, CA; www.illumina.com). All the data processing and analysis were performed using the algorithms included with the Bioconductor package beadarray and *Lumi* implemented in R software environment for statistical computing and graphics (R Foundation for Statistical Computing, Vienna, Austria; www.r-project.org).

### Microinjection of HPSC-derived NC cells and HPSC-derived endoderm cells in chicken embryos

For injections into the cardiac NC premigratory region, chicken (*Gallus domesticus*) eggs (Winter Egg Farm, Cambridge, United Kingdom) were incubated in a digital cabinet incubator (OVA Easy 380; Brinsea) for 32 h until Hamburger and Hamilton stage 9–10 (HH9–10). Eggs were windowed and injected under the embryo with India Ink to improve contrast. Small cuts were made with a BD Microlance needle (size 3) through the vitelline membrane and ectoderm directly adjacent to the neural tube, at a level just posterior to the forming otic vesicle. Clumps of 50–100 cells in Matrigel^™^ were injected into the cut site using a pulled glass capillary tube. Eggs were resealed with tape and cultured a further 20, 42, and 108 h after injection to visualize migrating NC cells at HH16, HH17, and HH26, respectively. HPSC-derived endoderm cells were used as a negative control and generated as previously reported [35].

For systemic injections into developing chicken embryo, eggs were incubated until HH24. A small window was made, and 500–1,000 NC cells were administered into the extraembryonic vessels. Either GFP^+^ HPSC-derived NC cells or GFP^+^ HPSC-derived endoderm cells were administered as previously reported [16,36]. The window was covered with parafilm (VWR), and eggs were placed horizontally in the incubator until HH34. Embryos were stained using whole mount immunocytochemistry as previously described [36].

### Differentiation of the NC to various cell types

NC populations were differentiated to SMC using a combination of PDGF-BB (10 ng/mL; Peprotech) and TGF-β1 (2 ng/mL; Peprotech), as previously reported [37]. NC differentiation to neuronal populations and MSC was performed as previously reported [21]. NC differentiation to melanocytes was performed as previously described [38]. MSC populations were subsequently differentiated to chondrocytes, adipocytes, and osteocytes using the StemPro Chondrogenesis Differentiation Kit (Thermo Fisher Scientific), StemPro Adipogenesis Differentiation Kit (Thermo Fisher Scientific), and StemPro Osteogenesis Differentiation Kit (Thermo Fisher Scientific), respectively, following the manufacturer's instructions.

### Cell proliferation assay

To assess cell proliferation, the CellTiter 96 Non-Radioactive Cell Proliferation Assay [[1] Kit was used, as per the manufacturer's instructions.

### Freezing and thawing of NC cells

Confluent NC cells were dissociated with TrypLE Express (Thermo Fisher Scientific). NC cells were pelleted and resuspended in CDM-PVA media. 2 × 10^6^ cells were added to a cryovial using 90% CDM-PVA media with 10% dimethyl sulfoxide (DMSO). NC cryovials were thawed at 37°C, and cells transferred to a 15 mL Falcon tube containing 8 mL of fresh CDM-PVA media. Cells were centrifuged for 3 min at 1,200 rpm. Cell pellets were resuspended in 1 mL FSB media, and 3 × 10^5^ NC cells were plated per well of a 0.1% gelatin-coated well of a six well plate. Cells were incubated at 37°C overnight in a 5% CO_2_ incubator. Cell viability was assessed as previously reported [39].

### Small interfering RNA knockdown and transient transfections

*TCOF1* messenger RNA (mRNA) was knocked down with small interfering RNA (siRNA; Thermo Fisher Scientific Assay ID S13920). siRNA transfection (25 nM) was performed using DharmaFECT-1 transfection reagent (Dharmacon) following the manufacturer's instructions.

### Generation of a TCOF1-targeting CRISPR guide RNA/Cas9 construct

A guide RNA (gRNA) targeting the TCOF1 gene was designed to target Exon1 according to the rule of 5′-GN20NGG-3′ (sequence 5′-TGGCTATGTGCGTGCGGCGC-3′). Oligonucleotides were synthesized and ligated into pSpCas9(BB)-2A-Puro (PX459) V2.0 as previously reported [40].

### Gene targeting

For gene targeting, 2.5 × 10^6^ HIPSC were electroporated with 1 μg of generated TCOF1 targeting Cas9 plasmid in 100 μL of nucleofection mix from the P3 Primary Cell 4D-Nucleofector X Kit (Lonza) using a 4D-nucleofector system (Lonza). Transfected cells were plated onto DR4 strain feeders (Jackson Laboratory) and cultured in advanced DMEM/F12 (Gibco) +20% KOSR supplemented with FGF2 (4 ng/mL) and Rho Kinase inhibitor (Y-27632, 10 μM). One day after transfection, cells were selected with puromycin (1 μg/mL; Sigma) for 36 h. Resistant colonies were picked and expanded, and mutation introduction was assessed by PCR and Sanger sequencing.

### Statistics

One-way analysis of variance (Tukey's multiple comparison test) and two-sided Student's *t*-test were used to determine statistically significant differences between the groups. Results are presented as mean ± standard error of the mean. *P* values ≤0.05 were considered statistically significant. All experiments represent the results of at least three independent biological replicates (measurements of biologically distinct samples). *P < 0.05; **P < 0.01; ***P < 0.001.

## Results

### Dissociation of differentiating neuroectoderm during early development promotes NC differentiation

It has been demonstrated that neuroectoderm can be generated from HPSC utilizing a combination of FGF2 and the TGF-β inhibitor SB-431542 (referred to herein as FSB) for 7 days [16]. Our group has previously reported the expression of some NC markers during this differentiation process, including *SNAI1*, *SNAI2*, and *PAX3*, suggesting that a mixed cell population may be generated at these stages [37].

We hypothesized that cell dissociation with trypsin, a serine protease, might facilitate EMT and the generation of the NC from this population [41–45]. We, therefore, differentiated the H9s HESC line to neuroectoderm over 7 days with FSB media, with and without WNT3A (25 ng/mL; R&D Systems).

**FIG. 1.**
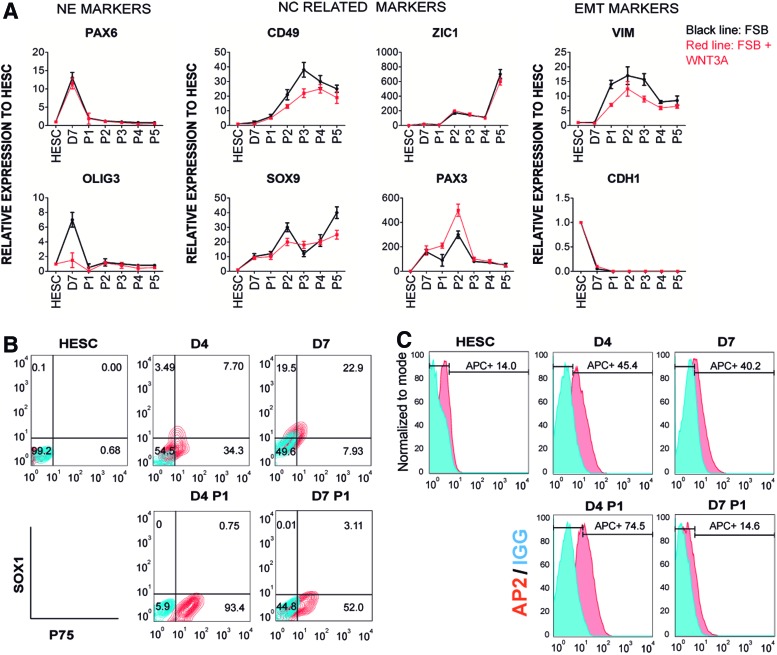

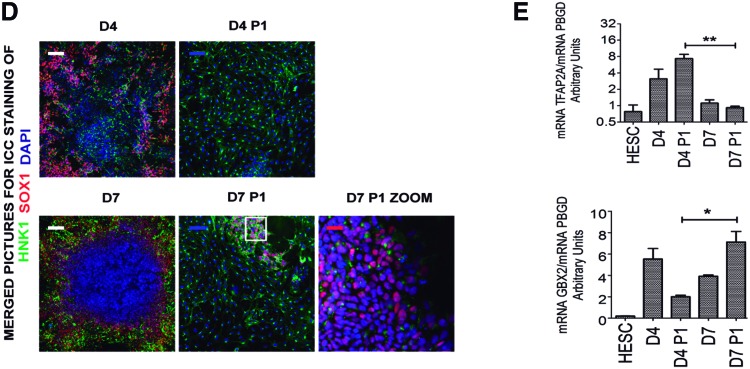
Differentiation of NC cells from HESC. **(A)** Expression of NE, NC, and EMT specific genes by qRT-PCR during H9s HESC differentiation in CDM, supplemented with FGF2, SB-431542 (FSB) and with (*red line*) or without WNT3A (*black line*). Cells were studied as undifferentiated HESC, NE day 7 (D7), and with serial passages (P1–P5). mRNA levels were normalized to the housekeeping gene *PBGD* and then presented relative to HESC expression which was set to 1. The results presented are representative of three independent experiments. **(B)** Flow cytometry analysis of NE marker SOX1 and NC marker P75 in the differentiation of H9s HESC to NE. FSB media was used to differentiate H9s HESC to NE over 4 or 7 days. SOX1 and P75 were analyzed before (D4, D7) and after (D4 P1 and D7 P1) splitting the NE at indicated time points. *Red contour* plots represent SOX1+P75 double stained populations, and *blue contour* plots represent IgG control staining. **(C)** Histogram of flow cytometric analysis of the NC marker TFAP2A (AP2) in H9s HESC differentiation with FSB to NE over 4 or 7 days. TFAP2A expression was measured before (D4, D7) and after (D4 P1, D7 P1) splitting the NE at indicated time points. *Red* histograms represent TFAP2A stained populations, and *blue* histograms represent IgG control staining. **(D)** Immunocytochemistry for the NC marker HNK1 and the NE marker SOX1 with DAPI counterstain in H9s HESC differentiation to NE. *Upper panels*, differentiation of HESC colonies over 4 days (D4) and after passage (D4 P1). *Bottom panels*, differentiation over 7 days. Colonies passaged at day 7 showed a remaining SOX1+ population (D7 P1, *white square*) shown in D7 P1 ZOOM. The SOX1+ population was not detected in colonies passaged on day 4. *White* scale bar: 100 μm, *blue* scale bar: 50 μm, *red* scale bar: 20 μm. **(E)** Expression of the NC marker *TFAP2A* and NE marker *GBX2* by qRT-PCR. H9s HESC colonies were differentiated with FSB media over 4 or 7 days to NE. RNA samples were collected at days 4 and 7 before and after passage (D4, D7, D4 P1, and D7 P1, respectively). The relative mRNA level was normalized to the housekeeping gene *PBGD*. The results are presented as mean ± SD of three independent experiments. *P < 0.05; **P < 0.01, two-sided Student's *t*-test. CDM, chemically defined media; EMT, epithelial–mesenchymal transition; FGF, fibroblast growth factor; HESC, human embryonic stem cell; mRNA, messenger RNA; NC, neural crest; NE, neuroectoderm; PBGD, porphobilinogen deaminase; qRT-PCR, quantitative real-time polymerase chain reaction; SD, standard deviation. Color images available online at www.liebertpub.com/scd

At day 7 of differentiation, the neuroectoderm was trypsinized and replated as single cells in CDM-PVA media supplemented with FGF2 and SB-431542 (FSB)±WNT3A for up to five passages. qRT-PCR over this period showed increased expression of NC differentiation-associated genes (*PAX3*, *ZIC1*, *CD49*, and *SOX9*) and the mesenchymal gene (*VIM*) (Fig. 1A). Conversely, neuroectoderm genes (*OLIG3* and *PAX6*) and an epithelial gene (*CDH1*) were downregulated (Fig. 1A), suggesting that cell dissociation promoted NC marker expression and EMT. Interestingly, the addition of WNT3A did not have any effect on the expression of these markers during the differentiation process (Fig. 1A).

To further analyze the onset of NC markers during the differentiation of HESC to neuroectoderm over the first 7 days, we assessed the expression of NC (*HNK1*, *P75*) and neuroectoderm (*PAX6*) genes on a daily basis. We observed the greatest expression of *HNK1* and *P75* on day 4 of the differentiation process (Supplementary Fig. S1A). Therefore, we hypothesized that dissociation of the developing neuroectoderm at day 4 instead of day 7 may increase the efficiency of NC generation during the differentiation process.

We analyzed SOX1 and P75 expression by flow cytometry in passaged and nonpassaged neuroectoderm at days 4 or 7 of differentiation in FSB. SOX1 is the earliest known specific marker of the neuroectoderm lineage and is activated during gastrulation [46]. Neuroectoderm passaged at day 4 (D4 P1) demonstrated a higher induction of P75 and lower expression of SOX1 compared with nonpassaged cells or cells split at day 7 (Fig. 1B). Furthermore, the cells split at day 4 attached and survived better than the cells split at day 7 (data not shown).

Consistent with these results, TFAP2A, an essential transcription factor for the development of the NC [47,48], was highly expressed in the D4 P1 population (Fig. 1C). SOX1 was detected at the edges of the neuroectoderm at days 4 and 7 of differentiation in FSB by immunocytochemistry (Fig. 1D). Following passage at day 4, SOX1 expression was significantly reduced. Conversely, SOX1 positive cells remained if passaging was delayed until day 7 (Fig. 1D), suggesting that the greatest reduction of SOX1 was obtained by passaging the differentiating neuroectoderm in FSB on day 4. Furthermore, HNK1 was highly expressed in passaged neuroectoderm at day 4 (Fig. 1D), and qRT-PCR analyses confirmed that these cells showed significantly higher levels of NC marker *TFAP2A* and lower levels of neuroectoderm marker *GBX2* [49] than cells from day 7 passaged neuroectoderm (Fig. 1E).

Together, these findings suggest that differentiation of neuroectoderm from HPSC can generate a mixed population containing both neuroectoderm and NC cells. The NC population can be enriched from the differentiating neuroectoderm by passaging at day 4 of the protocol.

### Serial passage increases purity of NC cells

**FIG. 2.**
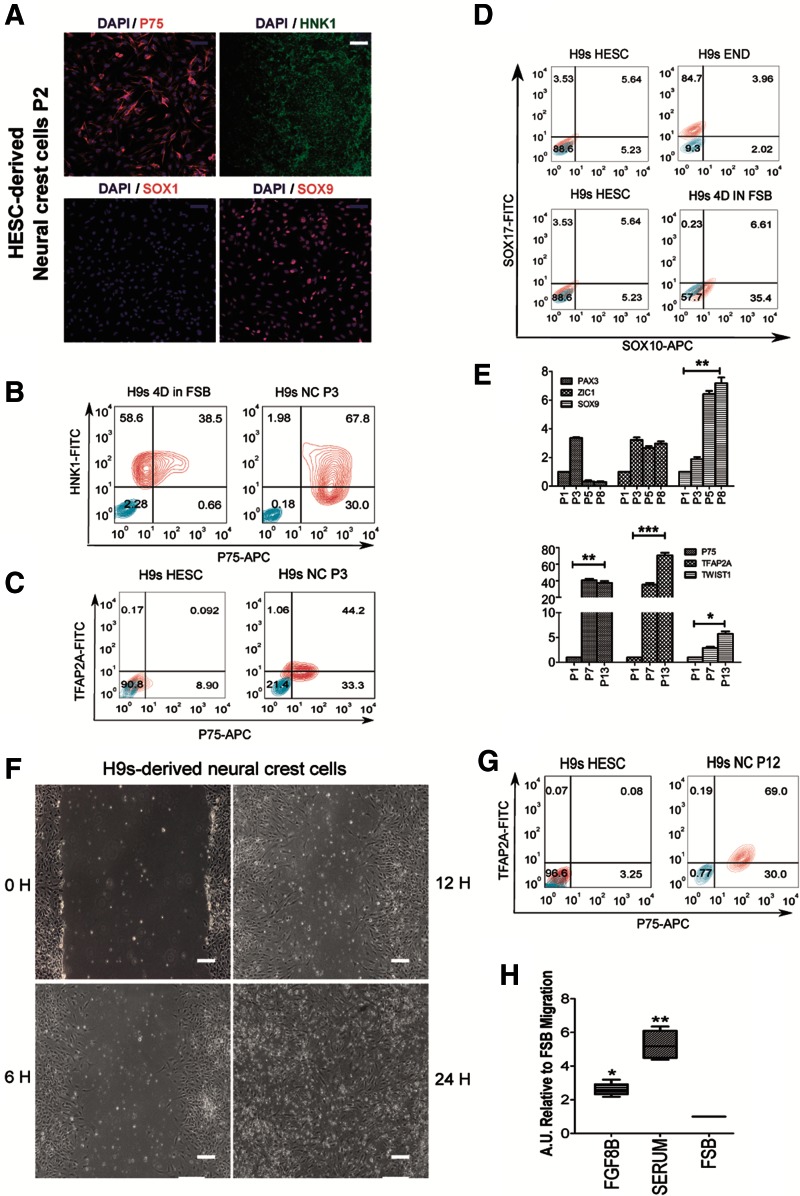
Increased purity and maturation of HESC derived NC cells with serial passages. **(A)** Immunostaining of the NC markers SOX9, P75, and HNK1 and the NE marker SOX1 with DAPI counterstain, in H9s HESC-derived NC after two passages (NC P2) in FSB media. *Blue* scale bar: 50 μm, *white* scale bar: 100 μm. **(B)** Flow cytometric analysis of P75 and HNK1 in H9s HESC after 4 days of differentiation in FSB media before passage (H9s 4D IN FSB) and H9s-derived NC after three passages maintained in FSB as single cells (H9s NC P3). *Red contour* plots represent HNK1+P75 double stained populations, *blue contour* plots represent IgG controls. **(C)** Flow cytometric analysis of TFAP2A and P75 in the differentiation of undifferentiated H9s and H9s-derived NC after three passages maintained in FSB as single cells (H9s NC P3). Colonies were passaged at day 4 of differentiation. *Red contour* plots represent TFAP2A+P75 double stained populations, *blue contour* plots represent IgG controls. **(D)** Flow cytometric analysis of SOX17 and SOX10 in H9s HESC, endoderm cells differentiated from H9s (H9s END), and H9s HESC at day 4 of differentiation in FSB without splitting (H9s 4D IN FSB). SOX10 induction was observed following 4 days of FSB treatment, whereas the population was negative for expression of the endoderm marker SOX17. *Red contour* plots represent SOX17+SOX10 double stained populations, and *blue contour* plots represent IgG controls. **(E)** *Top panel*: qRT-PCR expression analysis of NC markers *PAX3*, *ZIC1*, and *SOX9*. *Bottom panel*: qRT-PCR expression analysis of NC markers *P75*, *TFAP2A*, and *TWIST1.* mRNA of HESC-derived NC cells at different passages was used to perform the qRT-PCR. The relative mRNA levels were relative to NC passage 1 levels (NC P1). The relative mRNA level was normalized to the housekeeping gene *PBGD*. P1, P3, P5, P7, P8, and P13: number of passages of the NC cells as single cells. Results presented as mean ± SD of three independent experiments. *P < 0.05; **P < 0.01; ***P < 0.001. Two-sided Student's *t*-test. **(F)** Representative images of in vitro scratch wound assay performed in H9s HESC derived NC cells (NC P7). Pictures were obtained from 0 to 24 h postscratch. *White* scale bar: 100 μm. **(G)** Flow cytometric analysis of TFAP2A and P75 in H9s HESC and H9s HESC-derived NC passage 12 in FSB (NC P12). *Red contour* plots represent TFAP2A+P75 double stained populations, and *blue contour* plots represent IgG controls. **(H)** Chemotaxis potential and migration in representative H9s-derived NC P7 was assessed using a CytoSelect^™^ Transwell Cell Migration Assay Kit. The NC chemoattractant FGF8B was added to FSB media to assess migration. DMEM/F12 + 10% fetal bovine serum (SERUM) was used as a positive control of migration. FSB migration without any chemoattractant was set as 1, and data are expressed relative to this. Equal cell numbers were used in each condition, and results are presented as mean ± SD of three independent experiments. *P < 0.05; **P < 0.01. DMEM, Dulbecco's modified Eagle's medium. Color images available online at www.liebertpub.com/scd

Following passaging at day 4, cells were maintained in FSB medium upon reaching confluence (NC P1). Every 4 days, the confluent NC cells were split at a 1:3 ratio and seeded for the next passage (Supplementary Fig. S1B). After two passages cells expressed the NC proteins SOX9, HNK1, and P75 and did not express the neuroectoderm marker SOX1 (Fig. 2A). We detected expression of the NC markers P75 and TFAP2A by flow cytometry following subsequent passaging (Fig. 2B, C) [21]. While over 80% of the early NC population expressed P75, only 50% of the population was double positive for both P75 and TFAP2A at this stage (Fig. 2C). Furthermore, SOX10, a marker of migratory NC, was induced at D4 (Fig. 2D). These data confirmed that we were generating NC at this time point of the differentiation process, although the yield was suboptimal.

We questioned whether the differentiated NC would retain their NC-like identity following further expansion. Interestingly, the expression levels of the NC markers *PAX3* and *ZIC1*, markers of early NC induction [50,51], peaked at passage 3 (P3). In contrast, *SOX9*, which is an essential factor for the further development of migratory NC [52], was observed to be highly expressed at passage 8 (P8) (Fig. 2E, top). The expression levels of the NC markers *P75*, *TFAP2A*, and *TWIST1* increased significantly upon serial passaging (P1, P7, and P13), as assessed by qRT-PCR (Fig. 2E, bottom), suggesting that this approach could purify the NC population.

To further confirm that the NC cells retain their NC identity and show purification with passaging, we used flow cytometry to examine the expression of NC and non-NC markers. Furthermore, we repeated the flow analysis of P75 and TFAP2A in late passages of NC (P12) and observed that 99% of the cells expressed P75 and 69% showed both P75 and TFAP2A (Fig. 2G). Similar results were also obtained using HIPSC to generate NC cells (Supplementary Fig. S2A–C).

To assess if the purified NC population contained additional cell populations, we measured the expression of endoderm (*SOX17* and *EOMES*), mesoderm, (*NKX2.5* and *KDR*), and neuronal (*TUBB3* and *MAP2*) genes in the populations (Supplementary Fig. S2E). We observed negligible expression of these genes in the NC populations compared to HPSC-derived endoderm, mesoderm, or neurons, demonstrating that the NC did not contain a mixture of these populations in culture. Taken together, these data suggest that NC purification may be achieved by serial passaging of NC cells in FSB media. Furthermore, we found that we could expand the NC cells up to passage 15 without losing their NC identity [53].

### NC cells are proliferative and migrate in response to specific chemoattractants

To assess whether passage number or cell density affects the proliferation rate of the differentiated NC, we performed sequential cell counting and an MTT assay at different NC passages (Supplementary Fig. S2C, D). The MTT Cell Proliferation Assay is used to calculate the cell proliferation rate. The yellow tetrazolium MTT is reduced by active cells to purple formazan that can be solubilized and quantified by spectrophotometric means.

We found that the proliferation rate of NC was independent of passage number (Supplementary Fig. S2C) or cell density (Supplementary Fig. S2D). In vivo, NC cells migrate over great distances in response to chemotactic cues to contribute to tissue development during embryogenesis [54]. To validate a similar migratory function in our cells, we performed an in vitro scratch assay. NC cells were able to migrate and cover the scratch within 24 h (Fig. 2F). FGF8 is chemotactic and chemokinetic for NC in vivo and in vitro [19,54]. We, therefore, examined whether the HESC-derived NC cells would respond to this cue using the CytoSelect 24-well Cell Migration Assay (8 μm). In response to FGF8B, NC migration was significantly greater than in FSB media alone (Fig. 2H). Overall, these data demonstrate that the HESC-NC cells recapitulate critical NC functions.

### Early passages of HESC-NC comprise a mixed population of neural progenitors and premigratory NC cells that is purified with serial passage

**FIG. 3.**
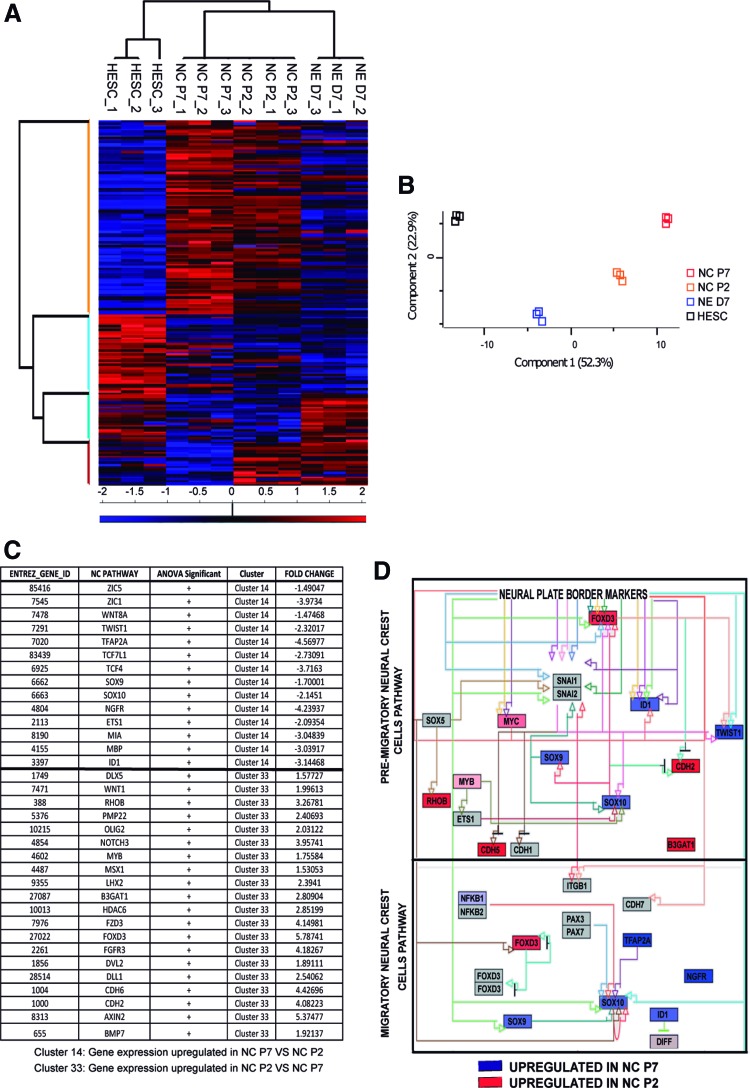
NC P2 cells are a mix of premigratory NC and neural progenitor cells that are purified into migratory NC cells with serial passages (P7). **(A)** Heat map showing the Euclidean distance metric among HESC, NC P2, NC P7, and NE D7 populations. The global gene expression patterns were sorted based on similarity by hierarchical clustering. *Red* (upregulation) and *blue* (downregulation) depict differential gene expression from the mean across all samples. NC P2, NC P7: H9s HESC treated 4 days with FSB then split as single cells for two or seven passages, respectively. NE D7: NE cells obtained from H9s HESC after 7 days in FSB. **(B)** Principal component analysis showed the NC P2 population as an intermediate population between NE and NC P7. **(C)** Fold change gene expression of NC and neural markers between NC P2 and NC P7 populations. Cluster 14 depicts the NC genes upregulated in NC P7 compared with NC P2. Cluster 33 illustrates the NC genes upregulated in NC P2 compared with NC P7. Benjamini–Hochberg FDR 0.05 was used as a cutoff for ANOVA of differentially expressed genes. **(D)** Gene interaction pathway for NC cell differentiation (WikiPathway WP2064 revision 47071). Differentially expressed genes are colored in *red* for upregulated gene expression in NC P2 or *blue* for upregulated gene expression in NC P7. PathVisio software was used to depict the NC pathway. Legend: *Arrows* represent stimulatory interaction, while T lines represent inhibition. The coloration intensity of the box is dependent on the level of differential expression between these two populations. Those which are highest in NC P7 are *blue*, and those highest in NC P2 are *red*. ANOVA, analysis of variance; FDR, false discovery rate. Color images available online at www.liebertpub.com/scd

To uncover the differences between early and later passages of NC, we performed a microarray gene expression analysis using H9s HESC and three independent differentiations of H9s HESC to neuroectoderm [34], NC at passage 2 (P2), and NC at passage 7 (P7). Hierarchical clustering separated these populations (Fig. 3A). Interestingly, PCA analysis distributed NC P2 in between neuroectoderm and NC P7 population (Fig. 3B). Based on this, we hypothesized that the early NC population could be a mixture of neuroectoderm and NC cells.

The NC P2 population expressed markers of neural progenitors, including *AXIN2* [55], *FGFR3* [56], and *NOTCH3* [57] (Fig. 3C). These genes, however, were markedly downregulated at later passages (Fig. 3C). Furthermore, the NC P2 population expressed markers of neural plate border/premigratory NC cells, including *CDH2* [58], *CDH6* [59], *FOXD3* [60,61], *BMP7*, and *RHOB* [9,62] (Fig. 3C). These neural plate border genes are thought to promote conversion of premigratory NC P2 to migratory cells as they also expressed migratory markers such as *B3GAT1* (HNK1) [63,64] in this mixed population (Fig. 3C).

As the WNT signaling pathway has been identified as playing important roles in NC development in vivo [23,65–67], we wondered why exogenous WNT3A was not required in our in vitro differentiation system. Interestingly, genes related to activation of WNT signaling, such as *WNT1*, *FZD3*, *DVL2* [68], and *MSX1* [69], were significantly expressed in the early NC P2 population (Fig. 3C). These data suggest that there may be endogenous activation of WNT during the early stages of NC differentiation, as observed in the neural plate border intermediate cells in vivo [69] and in vitro [23].

Interestingly, it has been proposed that NC induction requires intermediate levels of BMP signaling [70], as part of a BMP gradient between epidermal ectoderm which expresses both BMP7 and BMP4 [71] and the neural plate. BMP7 induces the expression of the RHOB protein in the cells destined to become NC in the neural plate border [9,62]. Consistent with developmental NC induction, *BMP7* and *RHOB* were highly upregulated in the early NC P2 population compared with NC P7. In contrast, the NC P7 population expressed higher levels of migratory NC genes such as *SOX10*, *SOX9*, *TWIST1*, and *P75* [52,58,72,73] compared to the early NC P2 population (Fig. 3C).

Finally, we plotted gene expression differences in the NC pathway using an open source pathway archive (WikiPathway WP2064 revision 47071). Most genes upregulated at NC P2 corresponded to genes expressed in premigratory NC cells (Fig. 3D). This population also showed some migratory NC genes, suggesting a mixed population. However, genes upregulated in the NC P7 population corresponded for the most part with genes expressed in the migratory NC (Fig. 3D). These data revealed evidence of endogenous WNT and BMP activity in the NC P2 population. In these early passages, a mixed NC population could be purified in vitro after several further passages to express a migratory NC transcriptional profile by passage 7.

### HESC-derived NC cells survive, engraft, migrate, and differentiate in vivo at NC specific location within the ascending aorta and the brain

NC cells should engraft in the appropriate locations for NC derivatives. In avian embryos, the fate of the NC is well established. The removal of the dorsal neural tube between the otic vesicle and the third somite in chicken embryos results in a variety of defects of the derivatives of the arch arteries [74]. Chicken embryos have previously been used as a host for studying the differentiation potential of human stem cells [36,75,76]. To demonstrate that the NC cells have the potential to migrate and differentiate to their specific locations in vivo and that freeze/thaw cycles do not affect the potential of the NC cells, we performed several experiments engrafting or injecting NC GFP^+^ cells into chicken developing embryos.

**FIG. 4.**
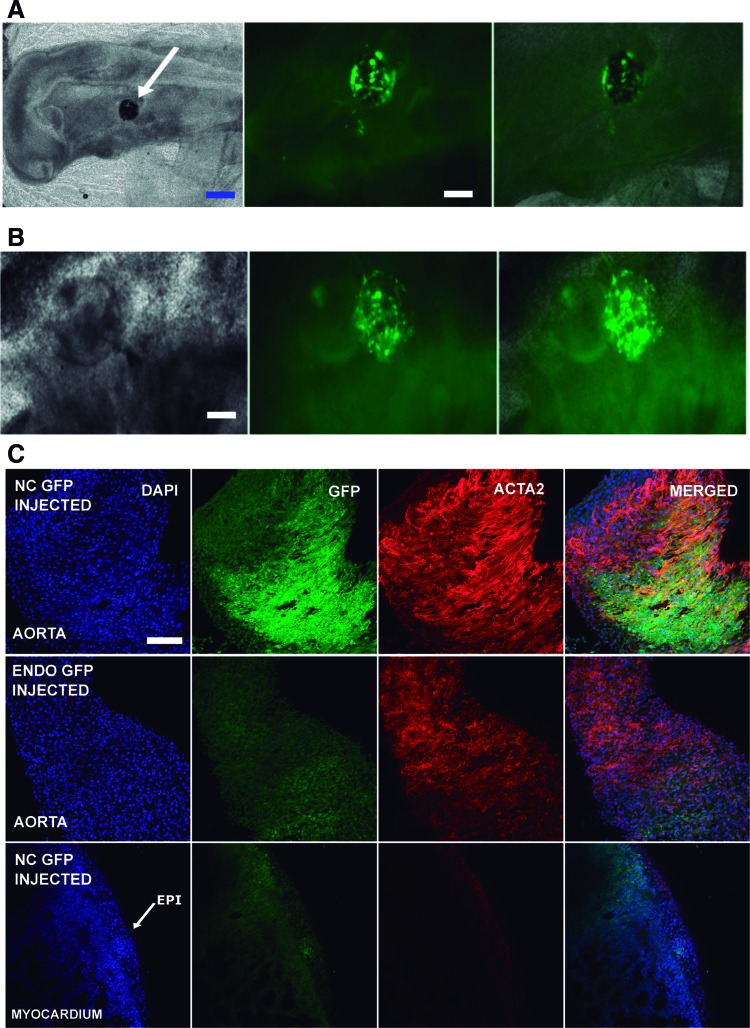
HESC-derived NC cells engraft, migrate, and differentiate into specific NC developmental location in the chicken embryo. **(A)** Fluorescent images of HESC-derived NC GFP^+^ cells showing survival into the chicken embryo, 20 h after engraftment at HH16. Clumps of 100–150 HESC-derived NC GFP^+^ cells at P2 were embedded into Matrigel^™^ and engrafted into the chicken embryo at HH9–10. The clumps were inserted in between the otic vesicle and the third somite, near to the neural tube (*white arrow*, *left panel*). *Blue* scale bar: 100 μm. *White* scale bar: 50 μm. **(B)** Fluorescent images showing HESC-derived NC GFP^+^ cells migrating from the Matrigel inside the embryo (HH17) 42 h after engraftment. *White* scale bar: 50 μm. **(C)** *Top row*: Confocal images from the chicken embryo aorta 4.5 days after HESC-derived NC GFP^+^ cell engraftment (HH29). The GFP^+^ cells migrated to the aortic arch (GFP^+^ picture) and differentiated into SMC (ACTA2 picture) contributing to the aortic wall. *Middle row*: Confocal images from chicken aorta 4.5 days after HESC-derived endoderm GFP^+^ cell insertion (HH29). No GFP^+^ cells were visible in the aortic arch of the chicken embryo when HESC-derived endoderm GFP^+^ cells were injected instead of the HESC-derived NC GFP^+^ cells. *Bottom row*: Confocal images from chicken myocardium and epicardium 4.5 days after HESC-derived NC GFP^+^ insertion (HH29). No GFP^+^ cells were identified in the epicardium or the myocardium of the chicken embryo. GFP^+^ cells were restricted to the aorta. *White* scale bar: 50 μm. HH, Hamburger and Hamilton; SMC, smooth muscle cell. Color images available online at www.liebertpub.com/scd

Fluorescent GFP^+^ HESC-derived NC P1 cells were thawed, harvested, and passed to NC P2 cells. Those cells were split, counted, and embedded into Matrigel for chicken embryo engraftment. Clumps of 50–100 cells were engrafted between the otic vesicle and the third somite, adjacent to the neural tube at HH9–10. Some embryos were harvested 20 and 42 h postengraftment (HH16–17) to monitor the success of the engraftment. Observation under an inverted epifluorescent microscope showed that they exhibited a lump of GFP^+^ cells in the region of the otic vesicle. Furthermore, migrating GFP^+^ cells were detected in all the embryos (Fig. 4A, B).

Four and a half days postengraftment (HH29), whole-mount confocal immunofluorescence imaging of the developing heart and ascending aorta was performed. A significant number of GFP^+^ cells were found in the ascending aorta expressing ACTA2 (Fig. 4C). Furthermore, GFP^+^ cells were not found in the aorta following engraftment of GFP^+^ HESC-derived endoderm embedded into Matrigel. In addition, we could not detect any NC-derived GFP^+^ cells in the epicardium or myocardium (Fig. 4C). Taken together, these data demonstrate that the engrafted human NC cells can migrate into the ascending aorta and contribute to the SMC population within the chicken embryo.

We also utilized an alternative method that we have previously validated for using chicken embryos as a host to study the differentiation and integration of HESC-derived cells [36]. GFP^+^ NC cells were injected (500–1,000 cells) into the extraembryonic circulation of chicken embryos at HH24. Embryos were harvested at HH34, and whole-mount confocal immunofluorescence was used for imaging of the brain, ascending aorta, and heart. We detected 10–20 GFP^+^ NC cells per embryo, around the ascending aorta (Supplementary Fig. S3A–F), and 40–50 GFP^+^ NC cells per embryo associated with the cerebral cortex vasculature (Supplementary Fig. S3G–J). We could not detect any human NC cells in the subepicardium neither the myocardium (Supplementary Fig. S3K), areas we have previously seen the localization of HESC-derived epicardial cells [36].

HESC-derived NC cells could be clearly discriminated from the host cells by their cell size, high green fluorescence (Supplementary Fig. S3A, D, G), and distinct nuclei (Supplementary Fig. S3B, E, H). Some of the engrafted NC cells around the ascending aorta also expressed ACTA2, suggesting the onset of EMT and SMC differentiation in situ within the ascending aorta (Supplementary Fig. S3C). Together, these results suggest that HESC-derived NC cells have access to ascending aorta in vivo and have the potential to contribute to tissue development in this location.

### In vitro differentiation of NC to a variety of cell types

NC cells can differentiate in vivo into a wide range of cell types such as neurons, melanocytes [77–79], MSC [80], adipocytes [81], chondrocytes, osteocytes [82,83], and vascular SMC [84]. We, therefore, followed a wide range of established differentiation protocols to confirm that our purified NC cells (NC P7 and further passages) were able to differentiate into their known derivatives.

**FIG. 5.**
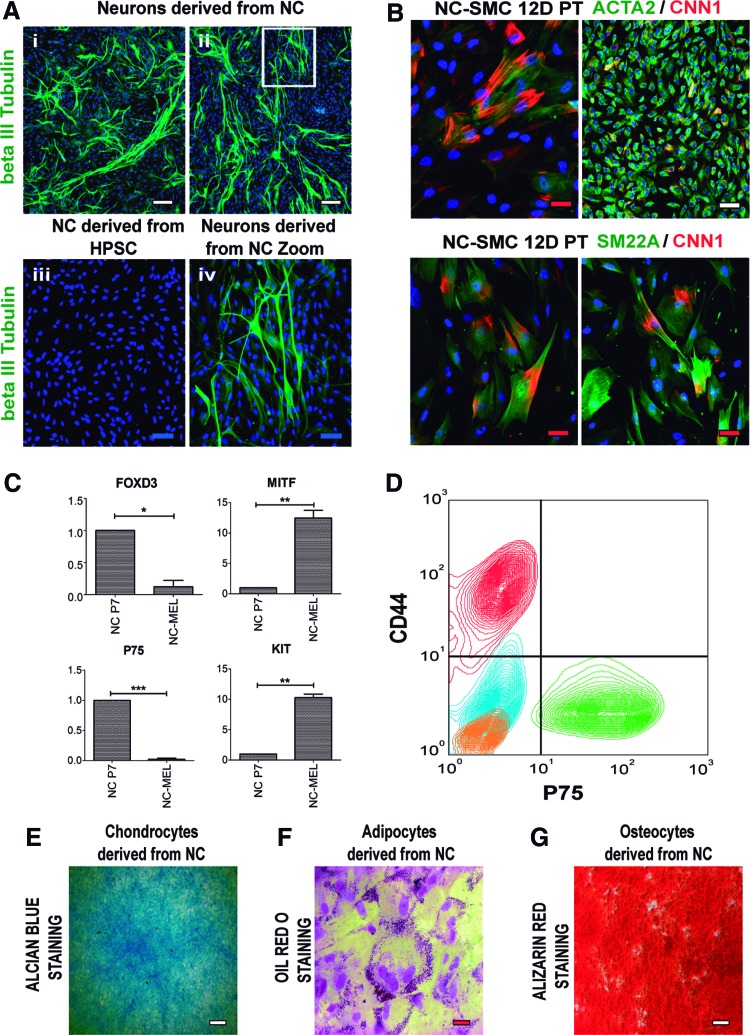
Differentiation of HESC-derived NC to their derivatives. **(A)** HESC-derived NC cells were plated on polyornithine-laminin-coated culture dishes to induce neuronal differentiation. **(A.i, A.ii)** Beta III Tubulin immunocytochemistry showing differentiation to peripheral neurons. **(A.iii)** NC cells stained with beta III Tubulin as a negative control. **(A.iv)** Magnification of the *white square* in **(A.ii)**. *White* scale bar: 100 μm, *blue* scale bar: 50 μm. Representative images of three independent biological replicates are shown. **(B)** Immunocytochemistry of the SMC proteins CNN1, ACTA2, and SM22A following HESC-derived NC differentiation to SMC with PDGF-BB (10 ng/mL) and TGF-β1 (2 ng/mL) for 12 days (12D PT). *White* scale bar: 100 μm, *red* scale bar: 20 μm. Representative images of three independent biological replicates. **(C)** qRT-PCR showing the upregulation of the specific melanocyte genes *MITF* and *KIT* and the downregulation of specific NC genes *FOXD3* and *P75* in NC P7 derived melanocytes (NC-MEL). The relative mRNA level was normalized to the housekeeping gene *PBGD*. Results are presented as mean ± SD of three independent experiments. *P < 0.05; **P < 0.01; ***P < 0.001. **(D)** Flow cytometry analysis of CD44 and P75 following differentiation of HESC-derived NC to MSC. *Green contour* plots represent CD44+P75 double staining in HESC-derived NCs, whereas *red contour* plots represent the double staining in MSC derived from this population. IgG isotype control staining for P75 and CD44 in both HESC-derived NC and MSC populations are shown as *blue* and *orange contour* plots, respectively. MSC derived from NC showed robust expression of CD44 and the absence of P75 expression. Representative results of three independent biological replicates are shown. **(E)** Alcian blue staining, **(F)** Oil Red O staining, and **(G)** Alizarin Red staining, demonstrating chondrocyte, adipocyte, and osteocyte differentiation from HESC-derived NC, respectively. Representative images of three independent biological replicates are shown. *White* scale bar: 100 μm, *red* scale bar: 20 μm. MSC, mesenchymal stem cells. Color images available online at www.liebertpub.com/scd

To assess the capability of the NC to differentiate into neurons, we plated cells on polyornithine-laminin-coated culture dishes [21]. After 10 days of differentiation, these cells spontaneously differentiated into beta III Tubulin^+^ neurons (Fig. 5A). Neuronal morphology was confirmed by microscopy (Supplementary Fig. S4B). Differentiation to SMC from NC was performed following our previous protocol [34]. SMC proteins such as ACTA2, SM22A, and CNN1 were detected by immunocytochemistry at 12 days of differentiation (Fig. 5B). *ACTA2*, *TAGLN*, and other SMC markers such as *MYH11*, *SMTN-B*, and *MYOCD* were also upregulated in differentiated SMC as assessed by qRT-PCR (Supplementary Fig. S4H).

Melanocyte differentiation was achieved following a previously reported protocol [38]. qRT-PCR assessed differentiation for melanocyte markers *KIT* and *MITF* [38] and NC markers such as *P75* and *FOXD3* (Fig. 5C). NC-derived melanocytes showed statistically significant upregulation of *KIT* and *MITF* expression and a marked downregulation of *P75* and *FOXD3*. Typical melanocyte morphology was confirmed by microscopy (Supplementary Fig. S4C). MSC were derived from the NC using a previously reported protocol [21]. Differentiated MSC was positive for CD44 and negative for P75 as assessed by flow cytometry (Fig. 5D) and expressed the MSC marker *CD105* (*ENG*) by qRT-PCR (Supplementary Fig. S4D) [85]. Chondrocytes, adipocytes, and osteocytes were successfully differentiated from NC using specific commercial differentiation media, following the manufacturer's instructions (Thermo Fisher Scientific).

Chondrocyte differentiation was demonstrated by Alcian Blue-positive staining of NC-derived chondrocytes (Fig. 5E). *ACAN* expression [86] also confirmed chondrocyte lineage (Supplementary Fig. S4E). Adipocyte differentiation was determined by positive oil red O staining (Fig. 5F) and *PPARG* [87] expression (Supplementary Fig. S4F). Osteocyte differentiation was shown by positive alizarin red staining (Fig. 5G). COL1A1 [88] and Osteocalcin [89] were detected by immunocytochemistry in osteocytes derived from NC (Supplementary Fig. S4G, top panel). Furthermore, the expression levels of the osteocyte genes sclerostin (*SOST*) and *COL1A1* were measured by qRT-PCR, revealing robust upregulation in NC-derived osteocytes (Supplementary Fig. S4G, bottom panel).

Finally, we examined the ability of these cells to function as NC cells after freeze–thawing. NC cells were frozen at passage 7 then defrosted and cultured for two further passages and retained their NC marker expression comparable to passage 9 NC cells that had not undergone freeze–thawing (Supplementary Fig. S4I, top panel). Similarly, freeze–thawing had no effect on the ability of the NC cells to generate derivatives such as SMC (Supplementary Fig. S4I, bottom panel). Furthermore, viability assays performed in thawed NC P2 and NC P7 cells [39] showed a recovery of 89% ± 6.5% and 92.5% ± 6.25%, respectively, from frozen vials (Supplementary Fig. S4J). The ability to freeze and store these cells is important for the practical utilization of these cells, which can now be bulked up for storage and used as required, without losing NC properties.

### Downregulation of Treacle expression by siRNA impairs cell migration in NC and MSC

Haploinsufficiency of TCOF1 in humans is associated with TCS, a condition characterized by craniofacial abnormalities thought to be due to impaired NC development [90]. To determine whether TCOF1 haploinsufficiency results in NC defects in humans, we initially investigated its role in HPSC-derived NC by siRNA-mediated knockdown. Transfection of TCOF1-targeted siRNA reduced mRNA expression in both NC and MSC as assessed by qRT-PCR (Supplementary Fig. S5A) and at the protein level in the NC by flow cytometry (Supplementary Fig. S5B).

**FIG. 6.**
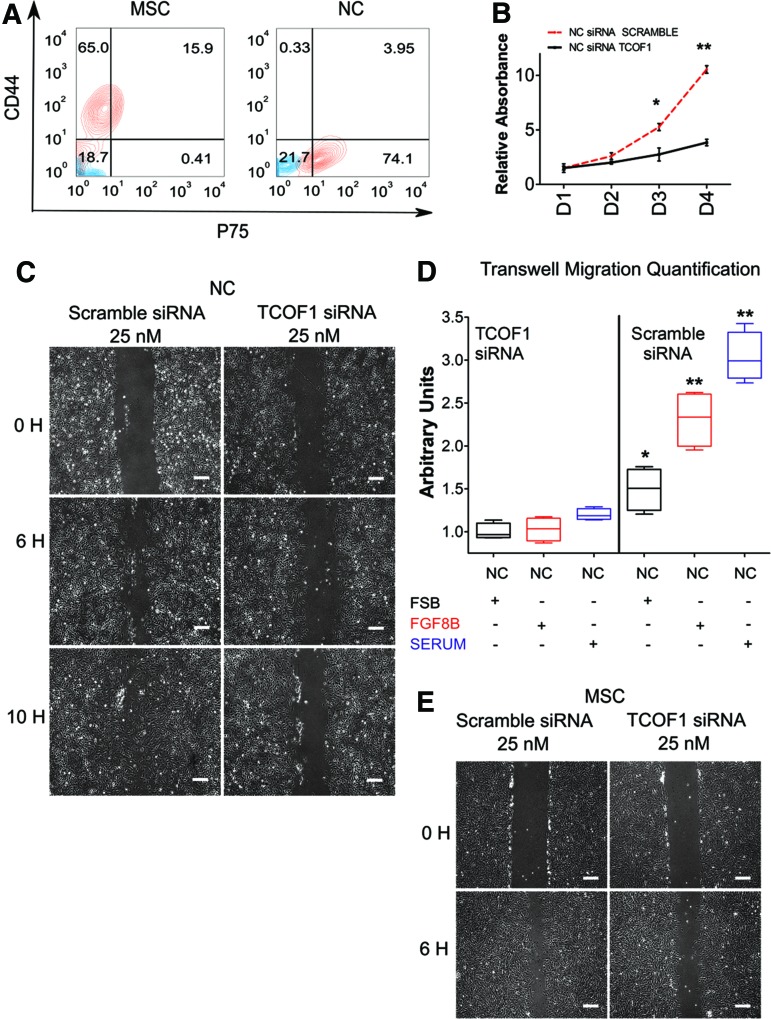
NC cells transfected with TCOF1 siRNA impair regular migration of NC and MSC. **(A)** H9s-derived NC cells were transiently transfected with a siRNA to TCOF1. *Left panel*: Flow cytometry was performed for CD44 and P75 following 5 days of differentiation from NC to MSC, demonstrating that TCOF1 KD does not impair MSC differentiation. *Right panel*: Flow cytometric analysis of CD44 and P75 in NC transiently transfected with a siRNA to TCOF1. *Red contour* plots represent CD44+P75 double stained populations, and *blue contour* plots represent isotype control staining. **(B)** MTT cell proliferation assay was performed using TCOF1 KD NC cells (NC siRNA TCOF1) and siRNA scramble NC cells (NC siRNA SCRAMBLE) during 4 days. Results are presented as mean ± SD of three independent experiments. *P < 0.05; **P < 0.01. Two-sided Student's *t*-test. **(C)** Representative images of scratch wound assays of HESC-derived NC cells transiently transfected with Scramble siRNA or TCOF1 siRNA. Images were collected 4 days following transfection, at the indicated time points. Scale bar: 100 μm. **(D)** *Box* plot depicting the quantification of chemotaxis potential and migration of NC cells transfected with TCOF1 siRNA or Scramble siRNA during a 6 h CytoSelect Cell Migration Assay. FGF8B was used as a NC chemoattractant. DMEM/F12 + 10% fetal bovine serum (SERUM) was used as a positive control for cell migration. Data are expressed relative to the NC cells transfected with TCOF1 siRNA, maintained in FSB medium. *P < 0.05; **P < 0.01. Two-sided Student's *t*-test. **(E)** Representative images of scratch wound assays of NC-derived MSC transiently transfected with Scramble siRNA or TCOF1 siRNA. Images were collected after 5 days of differentiation, at the indicated time points. Scale bar: 100 μm. KD, knockdown; MTT, 3-(4, 5-dimethylthiazolyl-2)-2, 5-diphenyltetrazolium bromide; siRNA, small interfering RNA. Color images available online at www.liebertpub.com/scd

To investigate whether TCOF1 has any role in the differentiation of NC to MSC, we differentiated NC P7 cells transfected with siRNA against TCOF1 or Scramble siRNA to a MSC fate as previously reported [21]. We observed no difference in the capacity for MSC differentiation from TCOF1 knockdown NC with upregulation of CD44 and downregulation of P75 observed in both knockdown and control conditions (Fig. 6A).

In mice, Treacle plays a role in NC development and proliferation [32]. We assessed if siRNA-mediated knockdown of TCOF1 impaired proliferation of HPSC-derived NC by performing an MTT assay. As in the mouse, we observed decreased proliferation in siRNA-mediated TCOF1 knockdown cells compared to controls (Fig. 6B).

It has previously been suggested that migration is not impaired in Tcof1^+/−^ mice [32]. To assess the role played by TCOF1 in human NC, we performed a wound healing scratch assay following siRNA-mediated knockdown of TCOF1. We observed impaired migration and scratch closure in TCOF1 knockdown NC compared to controls (Fig. 6C). Furthermore, while the migratory defect in this assay could be attributed to the documented impaired proliferation (Fig. 6B), the migration phenotype was detected as early as 6 h postscratch, suggesting that the phenotype was indeed due to impaired migration.

We performed further validation of this migratory defect using a CytoSelect transwell cell migration assay (Fig. 6D). siRNA-mediated knockdown of TCOF1 produced a significant reduction in NC migration compared with controls (Fig. 6D). This migration impairment was observed in FSB medium alone and observed in response to different NC chemoattractors such as FGF8B [19] or 10% fetal bovine serum (Fig. 6D). NC-derived MSC transfected with siRNA against TCOF1 also showed a similar migratory defect in a wound healing scratch assay (Fig. 6E). Taken together, these findings demonstrate that TCOF1 may play a role in both the proliferation and migration of the human NC.

### Modeling TCS in vitro with TCOF1^+/−^ HIPSC using CRISPR/CAS9 technology

**FIG. 7.**
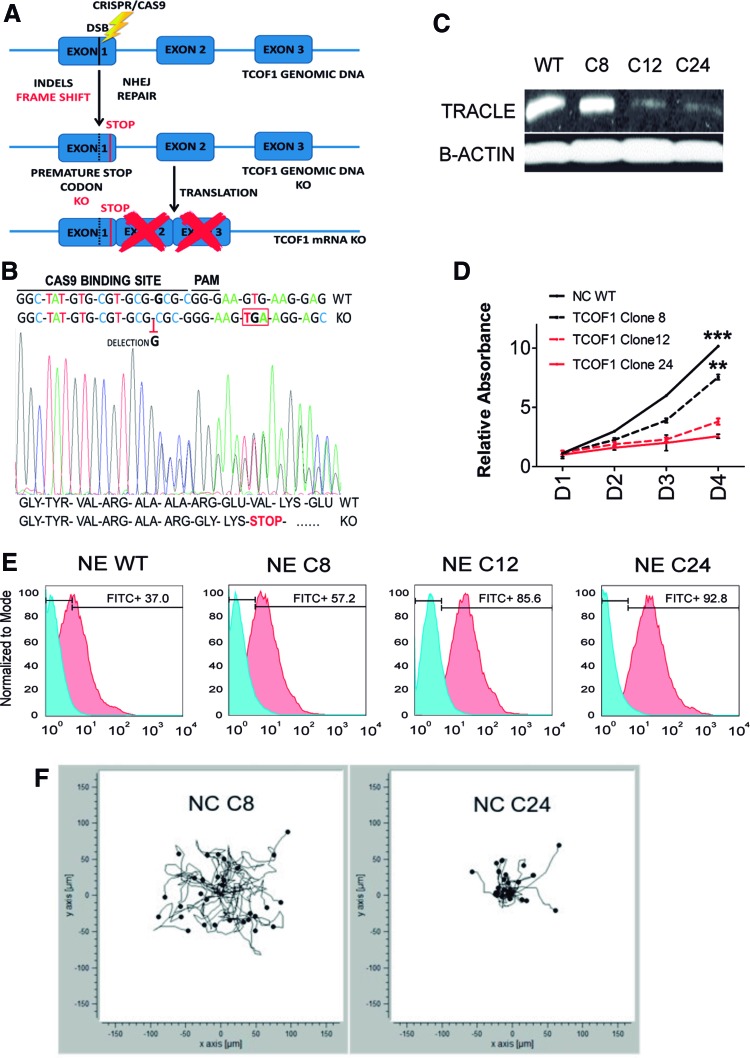
Generation of TCOF1 heterozygous knockout HIPSC using CRISPR/Cas9. **(A)** Schematic of CRISPR/Cas9-mediated nonhomologous end joining strategy to generate INDELs leading to HIPSC TCOF1 knockout clones. **(B)** Representative genomic sequencing of HIPSC transfected with specific CRISPR TCOF1 gRNA. Heterozygous knockout clones showed the same deletion leading to a change in codon reading frame and the introduction of a premature STOP codon in *TCOF1* Exon 1. **(C)** Immunoblot for Treacle protein demonstrating that NC derived from HIPSC TCOF1^+/−^ clones (C12 and C24) showed a reduction in Treacle compared with NC derived from HIPSC TCOF1^+/+^ (C8) and NC derived from H9s (WT). **(D)** Time course MTT proliferation assay of TCOF1^+/+^ NC derived from H9s (WT), HIPSC (C8), and NC derived from TCOF1^+/−^ HIPSC (C12 and C24), over a 4-day period. Proliferation rate decreased significantly in mutated cells compared with WT. Results are presented as mean ± SD of three independent experiments. **P < 0.01, ***P < 0.001, two-sided Student's *t*-test. **(E)** Flow cytometric analysis of Annexin V staining depicting the apoptotic rate in TCOF1^+/+^ NE-derived cells from H9s (WT), HIPSC (C8), and NE derived from TCOF1^+/−^ HIPSC (C12 and C24). *Red* histograms represent Annexin V staining, and *blue* histograms represent the unstained population. **(F)** Single cell analysis of cell migration in NC derived from TCOF1^+/+^ HIPSC (C8) and TCOF1^+/−^ HIPSC (C24). TCOF1^+/−^ NC demonstrates impaired migration and reduced directionality of movement. Each *dot* and *tail* represent a single cell analyzed, with 30 cells analyzed from both conditions, over a 12-h period. gRNA, guide RNA; HIPSC, human induced pluripotent stem cell; INDELs, insertions or deletions; WT, wild type. Color images available online at www.liebertpub.com/scd

To further confirm the results observed using TCOF1 siRNA in our NC model and to try to model TCS in vitro, we created heterozygous TCOF1^+/−^ KO HIPSC lines using CRISPR/Cas9 technology to accurately target Exon 1 of the TCOF1 gene in wild-type (WT) HIPSC (Fig. 7A). Cas9-induced double-strand breaks in TCOF1 genomic DNA were repaired by the nonhomologous end joining pathway leading to insertions or deletions (INDELs) in different HIPSC clones (Fig. 7B).

HIPSC Clones 12 and 24 (C12 and C24) had the same single nucleotide deletion in Exon 1 as observed by Sanger sequencing. This removal created a premature stop codon, resulting in a heterozygous KO for TCOF1 (Fig. 7B). HIPSC Clone 8 (C8) was also transfected, but no INDELs were detected in the TCOF1 gene (data not shown). We, therefore, used C8 as an isogenic WT HIPSC line to compare with the TCOF1^+/−^ clones (C12 and C24). NC was generated from all three clones, and NC P7 cells were used for subsequent studies. Immunoblotting for Treacle confirmed a significant reduction of protein levels in NC derived from TCOF1^+/−^ HIPSC clones (C12 and C24) compared with isogenic WT clone 8 NC and WT HESC-derived NC (Fig. 7C).

The downstream effect of defective ribosomal biosynthesis in mutant TCOF1^+/−^ mice is a lower proliferative index than WT NC cells [32]. TCOF1^+/−^ NC derived from clones C12 and C24 had significantly lower proliferation rate than similar WT NC cells derived from the C8 line and WT HESC, validating the findings observed in the TCOF1^+/−^ mouse (Fig. 7D).

The underlying cause of the reported craniofacial anomalies in the TCOF1^+/−^ mice is increased apoptosis in the neuroepithelium [32]. This developmental stage overlaps with the period of NC induction and migration from the neural plate border during embryogenesis [27]. To investigate whether our in vitro TCS model recapitulated this phenotype, we differentiated WT and TCOF1^+/−^ HIPSC lines to neuroectoderm [34]. Flow cytometric analyses for Annexin V confirmed a marked induction of apoptosis in neuroectoderm cells derived from TCOF1^+/−^ HIPSC (C12 and C24) compared with neuroectoderm derived from WT HIPSC Clone 8 or parental WT HESC (Fig. 7E).

As we unveiled a previously unreported migratory defect in NC and NC-derived MSC deficient for TCOF1 by siRNA (Fig. 6C, E), we performed the same analysis on the generated TCOF1^+/−^ lines. A wound healing scratch assay demonstrated impaired migration in both NC (Supplementary Fig. S6A) and MSC (Supplementary Fig. S6B) derived from TCOF1^+/−^ HIPSC C24. To further investigate this migratory defect uncovered by the wound healing scratch assay, we performed single cell analysis of cell migration and directionality of movement using real-time imaging of cells over a 12-h time course.

This analysis demonstrated that while WT HIPSC Clone 8 derived NC cells migrated freely in multiple directions over a 12-h period, TCOF1^+/−^ HIPSC derived NC cell shows considerable impairment of migration at a single cell level (Fig. 7F). These findings further validate the reduced migration phenotype of TCOF1-deficient NC cells in the human context.

## Discussion

This study first describes a simple and efficient method of generating NC cells from HPSC using CDM. Importantly, this in vitro system has great potential for investigating the molecular and cellular defects in neurocristopathies such as TCS. We have established a model of TCS using CRISPR/Cas9 gene editing and identified novel NC and MSC migration defects in our in vitro model.

### Development of a differentiation protocol to generate NC from HPSC using a CDM

Several methods have been reported to produce NC from HPSC, although further work is required to allow the production of cells in a fully CDM and to reduce the complexity of the differentiation conditions. The limitations of the previously reported protocols include the use of biological substrates such as Geltrex^™^ [21] or Matrigel [23] that display original batch to batch variability. Other protocols used media enriched with complex supplements such as N2^™^ and B27^™^ [23,43].

We have demonstrated that we can generate NC cells from HPSC using minimal cytokines compared with previous protocols [18–21] or WNT pathway activation [23]. This reduction of cytokines makes our protocol simpler and more economically viable. Furthermore, we do not need to perform cell sorting to generate our populations, as we have observed that serial passaging effectively purifies the populations generated.

We have also demonstrated that our cells can undergo freeze–thawing with up to 90% recovery rates and viability, permitting these cells to be used to generate stocks of NC cells at the same passages (Supplementary Fig. S4J). We have used thawed NC cells for multiple differentiations and have not seen any alterations in their maintenance or differentiation capacity following thawing. Therefore, our protocol permits the generation of a high number of NC cells which can be banked and used at later dates.

The differentiation of NC from HPSC using this approach recapitulates, to some extent, the normal embryonic developmental stages. In our protocol, the passage of neuroepithelial neuroectoderm at low density in FSB appeared to select the NC population in vitro. The genome-wide mRNA expression studies suggested that earlier passages of NC populations were a mixed population of neural progenitor cells and premigratory NC cells. Premigratory NC genes such as *RHOB*, *CDH6*, and *FOXD3* and neural progenitor genes such as *AXIN2* [55], *LHX2* [91], *DVL2* [92], and *OLIG2* [93] were upregulated in these populations (Fig. 3B).

It has been previously reported that the patterning of neural versus NC lineages is based on the plating density of early progenitors [18]. Serial passage of our cells at low density selected against the neural population with a significant downregulation of neural genes by later passages (P7). Meanwhile, the P7 cells expressed markers of migratory NC cells, including *SOX9*, *SOX10*, *P75*, and *TWIST*, and downregulated premigratory markers such as *WNT1*, *PAX3*, and *RHOB*.

Further validation of in vitro selection upon serial passaging is provided by the pattern of expression of *SOX10* and *WNT1*. In the mouse, *Sox10* expression follows *Wnt1* expression and marks virtually all NC cells immediately after their delamination from the neural tube [94]. In our system, *WNT1* is lowly expressed in mixed NC population, while it increases alongside *SOX10* expression in further passages, confirming NC purification in vitro with passaging.

In contrast to other in vitro protocols [18,19,21,43], we did not need to modulate WNT or BMP signaling exogenously. We hypothesized that the endogenous activation of WNT and BMP signaling, as evidenced by our microarray analysis, was sufficient in early passages to induce NC differentiation. This endogenous signaling may reflect the cross-talk and local gradients between a mixed population of the neural plate and non-neural plate ectoderm. Upon further passage of the mixed NC P2 population, we observed downregulation of both BMP and WNT signaling cells in these populations, suggesting that the combination of endogenous BMP and WNT signaling at the early stages of the differentiation protocol was sufficient to specify NC differentiation. The protocol imitates, in part, the steps involved in the development of NC cells in the embryo [9].

The properties of NC cells generated with this protocol were broadly characterized both in vitro and in vivo. We demonstrated the expression of NC markers from passage 7 to beyond. NC cells could proliferate, self-renew, and migrate to appropriate cues such as FGF8B, which has previously been reported [54]. Furthermore, we successfully demonstrated that these NC cells differentiate into their derivative lineages, including MSC, melanocytes, peripheral neurons, SMC, adipocytes, chondrocytes, and osteocytes (Supplementary Fig. S4) [21,23,95].

Importantly, in vivo characterization provides a critical test of a progenitor population's developmental capacity. To determine whether HPSC derived NC cells demonstrated functionality in vivo, we evaluated the integration and migration of these cells in the developing chick embryo. We found the engrafted NC cells localized in the wall of the ascending aorta (Fig. 4), the aortic arch, and the meningeal vessels of the brain (Supplementary Fig. S3).

The absence of NC cells in the epicardium and subepicardial regions of the heart confirmed the specificity of these cells in localizing correctly to NC in vivo developmental location. There could be several factors that mediate the migration and localization of human NC cells in vivo, including paracrine signals from chicken NC-derived tissues or cell–cell and cell–matrix interactions at particular NC-derived locations.

While we have demonstrated that our HIPSC-derived NC can contribute to the aortic SMC population in vivo, we have not explored the broad differentiation of these cells using this approach. The ability to transplant HIPSC-derived cells in vivo provides an opportunity to further our understanding of the developmental potential of human progenitor cells within an in vivo context. Analysis of NC, neuron/glial, and pericyte marker expression in the transplanted cells may provide additional insight into the differentiation potential of these cells in vivo and their cell fate.

### Modeling Treacher Collins syndrome in vitro

The ability to generate NC from patient-derived HIPSC lines provides a unique tool to study neurocristopathies in the affected cell lineage. A complementary approach to study specific diseases is the de novo creation of a particular mutation in WT lines of HPSC using CRISPR/Cas9 gene editing. This method permits the investigation of multiple disease causing mutations, without the requirement of patient tissue. Moreover, this approach removes the influence played by various genetic backgrounds from different patient samples.

A vast number of NC-related diseases have been reported in the literature, including TCS, although studies using human models are lacking. Treacle protein depletion has been attempted previously using knock-in of a destabilization domain-tagging TCOF1 in HEK293 cells, resulting in impaired cell proliferation [87]. Furthermore, mice haploinsufficient for Treacle have been reported and demonstrate robust developmental NC phenotypes [32].

To finally confirm the quality and utility of the HPSC-derived NC using this protocol we attempted to model TCS in vitro. We created TCOF1^+/−^ HIPSC lines from WT cells using CRISPR/Cas9 technology. The differentiation of TCOF1^+/−^ HIPSC clones with our protocol provided the opportunity to study the functionality of the affected NC cells. TCOF1^+/−^ HIPSC derived NC cells displayed significant depletion of Treacle protein, and their phenotype corresponded to the mutant phenotype found in the TCOF1^+/−^ mouse model [32].

Furthermore, neuroectoderm derived from TCOF1^+/−^ HIPSC showed a dramatic increase in apoptosis, replicating the aberrant cell death in the neural plate in TCOF1^+/−^ mice [32]. The haploinsufficiency of Treacle also leads to reduced proliferation of NC cells in TCOF1^+/−^ mice [32]. NC derived from TCOF1^+/−^ HIPSC also showed a significant reduction in proliferation compared with WT HIPSC-derived NC cells. Strikingly, we also demonstrated a previously unappreciated impairment in the migration of both TCOF1^+/−^ NC and NC-derived MSC in our in vitro model. Models of TCS have been reported in both the zebrafish and the mouse [32,96]. Both systems demonstrate extensive craniofacial abnormalities, yet the developing NC populations are shown to migrate and populate the maxillary and frontonasal regions normally.

It is of interest to note that while there is no reported difference in the migration behavior of the NC, there is an overall reduction in the number of migrating NC cells, and this has been demonstrated to be due to a proliferation and cell death defect at the early stages of NC delamination from the neuroepithelium. However, it is possible that the finding of fewer migrating NC cells in this model may also be due to a proportion of cells which fail to migrate, in addition to the clear cell survival defects reported [32,97]. While we report a migration defect in our TCOF1^+/−^ HIPSC-based NC system, it is important to acknowledge that this is an in vitro model and so is limited by the many missing developmental cues and triggers which are present in vivo. Therefore, it is possible that the migratory phenotype we report in this study may not be recapitulated during human in vivo development.

Further engraftment studies using TCOF1^+/−^ HIPSC-derived NC within the cranial NC may provide novel insights into the mechanisms involved during the migration of the human NC and their contribution to cells of the facial prominences and subsequent craniofacial compartments. Nevertheless, our TCOF1^+/−^ HIPSC-based NC model now adds to the limited information available regarding human NC development and its contribution to TCS.

## Conclusion

We believe that this protocol will serve as a resource for researchers seeking to produce NC and NC-derived tissues and to model neurocristopathies in vitro. Furthermore, we have shown that NC cells derived from HPSC can be differentiated into a wide variety of cell lineages which are of great interest to the field. The ability to study the mechanisms of NC biology and disease should also accelerate the development of innovative therapies to treat, or even prevent, NC disorders such as TCS.

## Supplementary Material

Supplemental data

Supplemental data

Supplemental data

Supplemental data

Supplemental data

Supplemental data

Supplemental data

Supplemental data
