# Supplementary material for: A Novel Human Pluripotent Stem Cell-Derived Neural Crest Model of Treacher Collins Syndrome Shows Defects in Cell Death and Migration
Source: Stem Cells Dev. 2019 Jan 10;28(2):81–100. doi: 10.1089/scd.2017.0234 (PMC6350417; doi:10.1089/scd.2017.0234)

Supplementary Table S1. Quantitative Real-Time Polymerase Chain Reaction Primers Used to Measure the Expression Levels of the Genes Listed

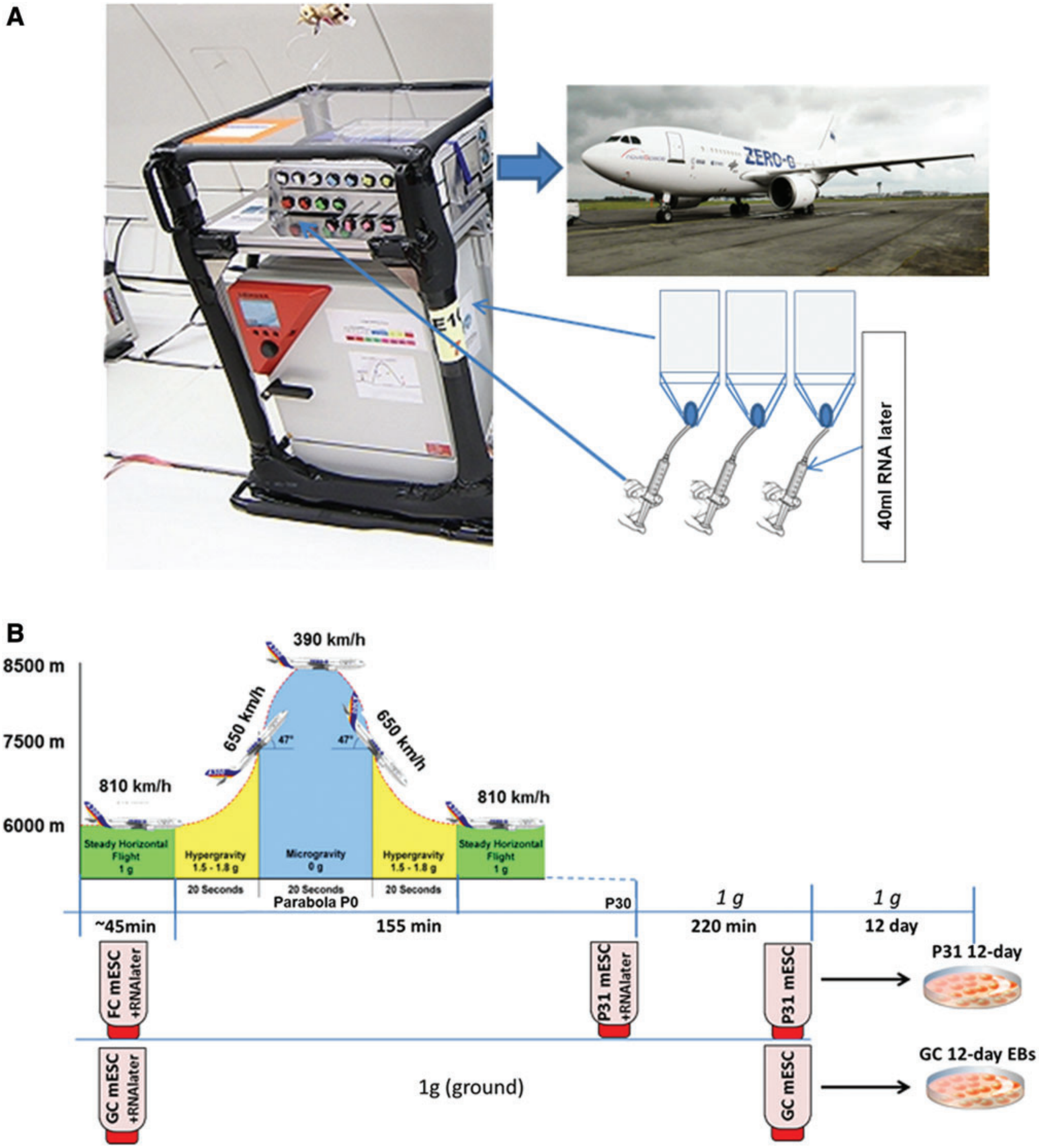

Supplement: Supplemental data [file Supp_Table1.pdf]
