# Supplementary material for: A Novel Human Pluripotent Stem Cell-Derived Neural Crest Model of Treacher Collins Syndrome Shows Defects in Cell Death and Migration
Source: Stem Cells Dev. 2019 Jan 10;28(2):81–100. doi: 10.1089/scd.2017.0234 (PMC6350417; doi:10.1089/scd.2017.0234)
Supplement: Supplemental data [file Supp_Table2.pdf]

Supplementary Table S2. Antibodies Used in Immunofluorescence, Western Blot, and Flow Cytometry Assays

ANTIBODY	Manufacturer	Reference	Source
**ANTI-BETA III TUBULIN**	AbCam	AB18207	Rabbit
**ANTI-AP2 ALPHA [EPR2688(2)]**	AbCam	AB108311	Rabbit
**ANTI-BETA-ACTIN**	Sigma	A1978	Mouse
**ANTI-CALPONIN**	Sigma	C2687	Mouse
**ANTI-CD44**	BD Phamingen	555478	Mouse
**ANTI-COLLAGEN I ALPHA 1**	R&D Systems	AF6220	Sheep
**ANTI-HNK1**	Sigma	C6680-100TST	Mouse
**ANTI-NGFR P75 (C-20)**	Santa Cruz	SC-6188	Rabbit
**ANTI-NGFR P75-ALEXA FLUOR 647**	BD Pharmingen	560326	Mouse
**ANTI-OSTEOCALCIN**	R&D Systems	MAB1419	Mouse
**ANTI-SM22A**	AbCam	AB14106	Rabbit
**ANTI-SOX1**	R&D Systems	AF3369	Goat
**ANTI-SOX10 [2E7B5]**	AbCam	AB181466	Mouse
**ANTI-SOX17**	AbCam	AB84990	Mouse
**ANTI-SOX9**	AbCam	AB185966	Rabbit
**ANTI-TCOF1**	Abnova	H00006949-M02	Mouse
