# Supplementary material for: A Novel Human Pluripotent Stem Cell-Derived Neural Crest Model of Treacher Collins Syndrome Shows Defects in Cell Death and Migration
Source: Stem Cells Dev. 2019 Jan 10;28(2):81–100. doi: 10.1089/scd.2017.0234 (PMC6350417; doi:10.1089/scd.2017.0234)
Supplement: Supplemental data [file Supp_Fig1.pdf]

Supplementary Data

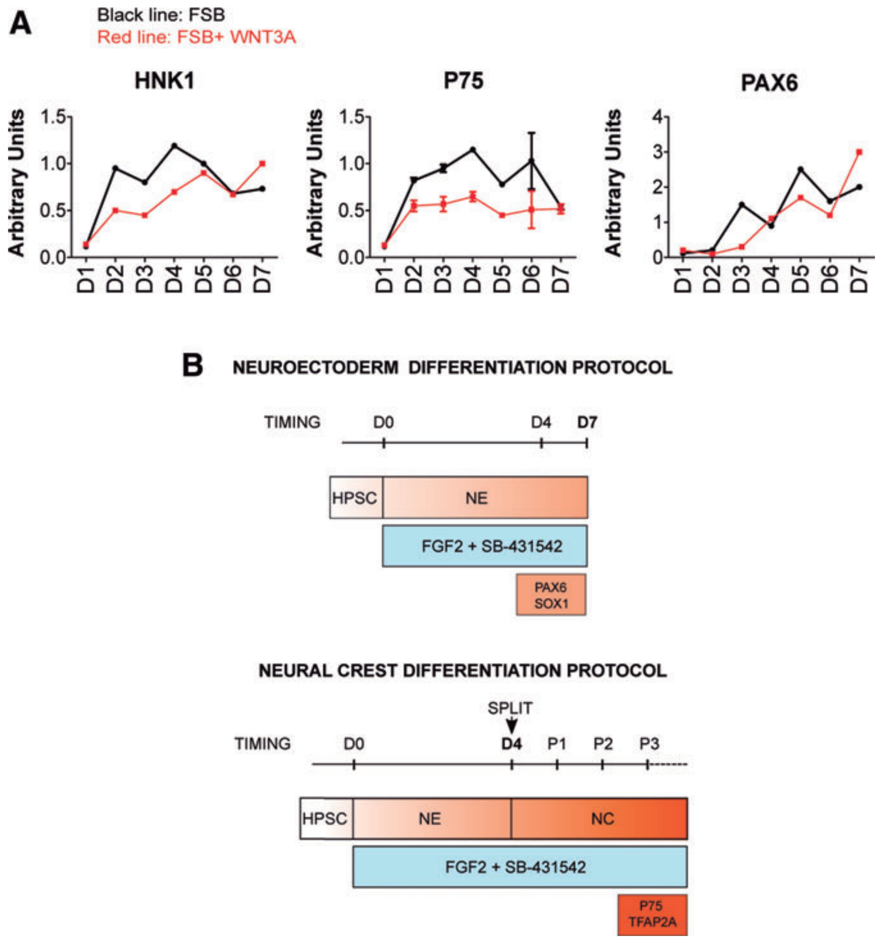

SUPPLEMENTARY FIG. S1. Optimization of NC cell differentiation by splitting NE-derived HPSC at day 4 into single cells. **(A)** Expression levels of *P75*, *HNK1*, and *PAX6* by qRT-PCR during 7 days of HESC differentiation to NE. The mRNA levels were normalized to the housekeeping gene *PBGD*. *Black line*: differentiation to NE with CDM supplemented with FGF2 and SB-431542 (FSB). *Red line*: FSB media supplemented with 25 ng/mL WNT3A. **(B)** Schematic of the differentiation protocols used to derive NE and NC cells from HPSC. CDM, chemically defined media; FGF, fibroblast growth factor; HESC, human embryonic stem cell; HPSC, human pluripotent stem cell; mRNA, messenger RNA; NC, neural crest; NE, neuroectoderm; PBGD, porphobilinogen deaminase; qRT-PCR, quantitative real-time polymerase chain reaction.
