# Supplementary material for: A Novel Human Pluripotent Stem Cell-Derived Neural Crest Model of Treacher Collins Syndrome Shows Defects in Cell Death and Migration
Source: Stem Cells Dev. 2019 Jan 10;28(2):81–100. doi: 10.1089/scd.2017.0234 (PMC6350417; doi:10.1089/scd.2017.0234)
Supplement: Supplemental data [file Supp_Fig2.pdf]

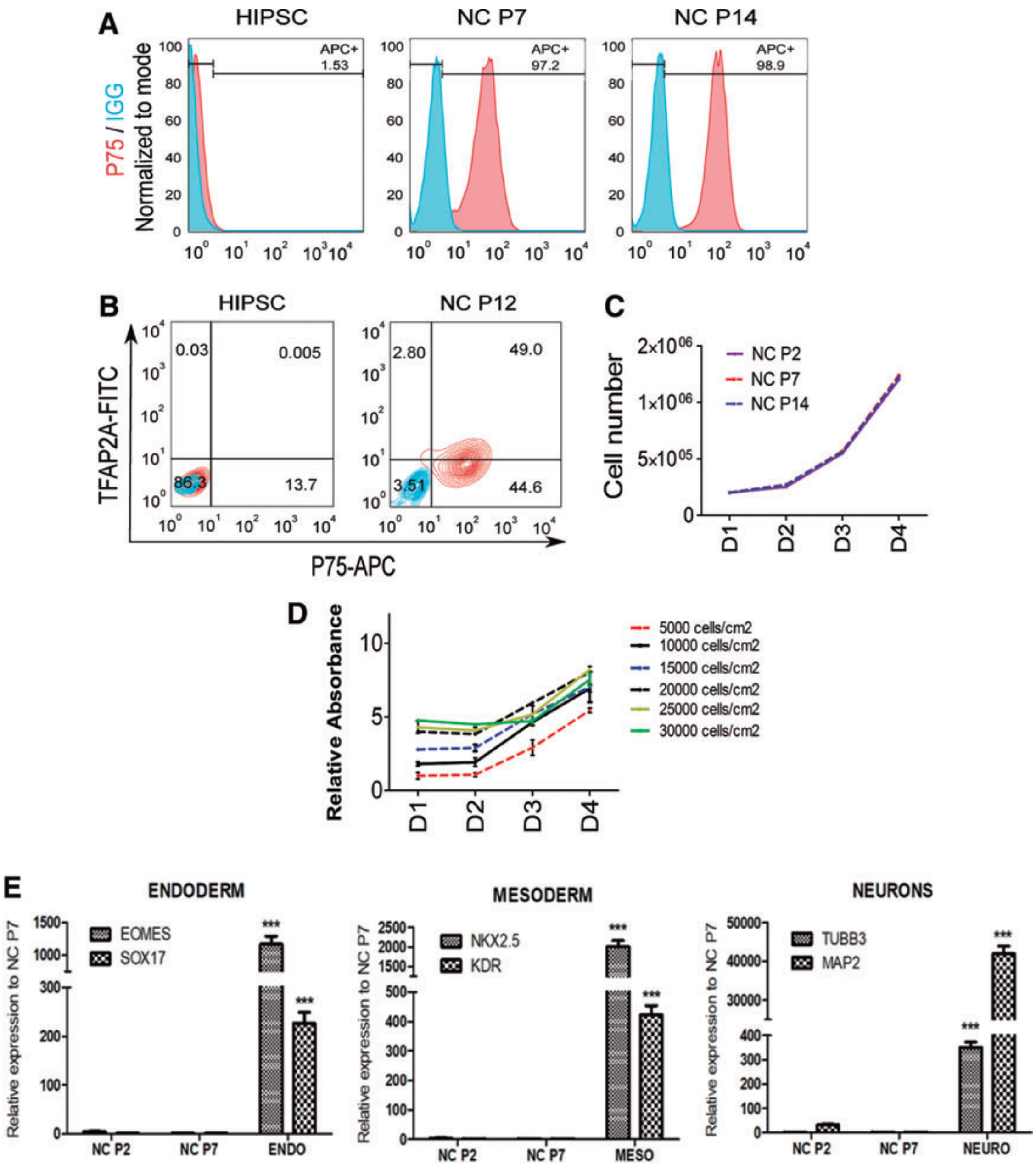

SUPPLEMENTARY FIG. S2. Characterization of NC cells derived from HIPSC. **(A)** Histogram of the percentage of P75+ cells in HIPSC (*left panel*), HIPSC-derived NC P7 (*middle panel*), and HIPSC-derived NC P14 (*right panel*) determined by flow cytometry. *Red* histograms represent P75 stained populations, *blue* histograms represent IgG controls. **(B)** Flow cytometry analysis of TFAP2A and P75 expression in wild-type HIPSC and their NC-derivatives (NC P12). *Red contour* plots represent TFAP2A+P75 double stained populations, and *blue contour* plots represent IgG controls. **(C)** Cell counts performed over a 4-day period from HIPSC-derived NC at P2, P7, and P14. The doubling time of the NC cells was 17.0 ± 0.71 h. **(D)** MTT proliferation assay performed on HIPSC-derived NC over a 4-day period in culture. Cells were seeded at various densities as depicted in the figure. Results are presented as mean ± SD of three independent experiments. **(E)** qRT-PCR analysis of endoderm (*EOMES* and *SOX17*), mesoderm (*NKX2.5* and *KDR*), and neuronal (*TUBB3* and *MAP2*) gene expression in NC P2 and NC P7 populations. The relative mRNA level was normalized to the housekeeping gene *PBGD*, and expression levels are relative to those at NC P7. The results are presented as mean ± SD of three independent experiments. ***$P < 0.001$, two-sided Student's *t*-test. ENDO, endoderm cells differentiated from HPSC [1]; MESO, mesoderm cells differentiated from HPSC [2]; NEURO, neurons differentiated from HPSC [3]. HIPSC, human induced pluripotent stem cell; SD, standard deviation.

Supplementary References

1. Yiangou L, ADB Ross, KJ Goh and L Vallier. (2018). Human pluripotent stem cell-derived endoderm for modeling development and clinical applications. Cell Stem Cell 22:485–499.
2. Cheung C, AS Bernardo, RA Pedersen and S Sinha. (2014). Directed differentiation of embryonic origin-specific vascular smooth muscle subtypes from human pluripotent stem cells. Nat Protoc 9:929–938.
3. Pawlowski M, D Ortmann, A Bertero, JM Tavares, RA Pedersen, L Vallier and MRN Kotter. (2017). Inducible and deterministic forward programming of human pluripotent stem cells into neurons, skeletal myocytes, and oligodendrocytes. Stem Cell Rep 8:803–812.
