# Supplementary material for: A Novel Human Pluripotent Stem Cell-Derived Neural Crest Model of Treacher Collins Syndrome Shows Defects in Cell Death and Migration
Source: Stem Cells Dev. 2019 Jan 10;28(2):81–100. doi: 10.1089/scd.2017.0234 (PMC6350417; doi:10.1089/scd.2017.0234)
Supplement: Supplemental data [file Supp_Fig3.pdf]

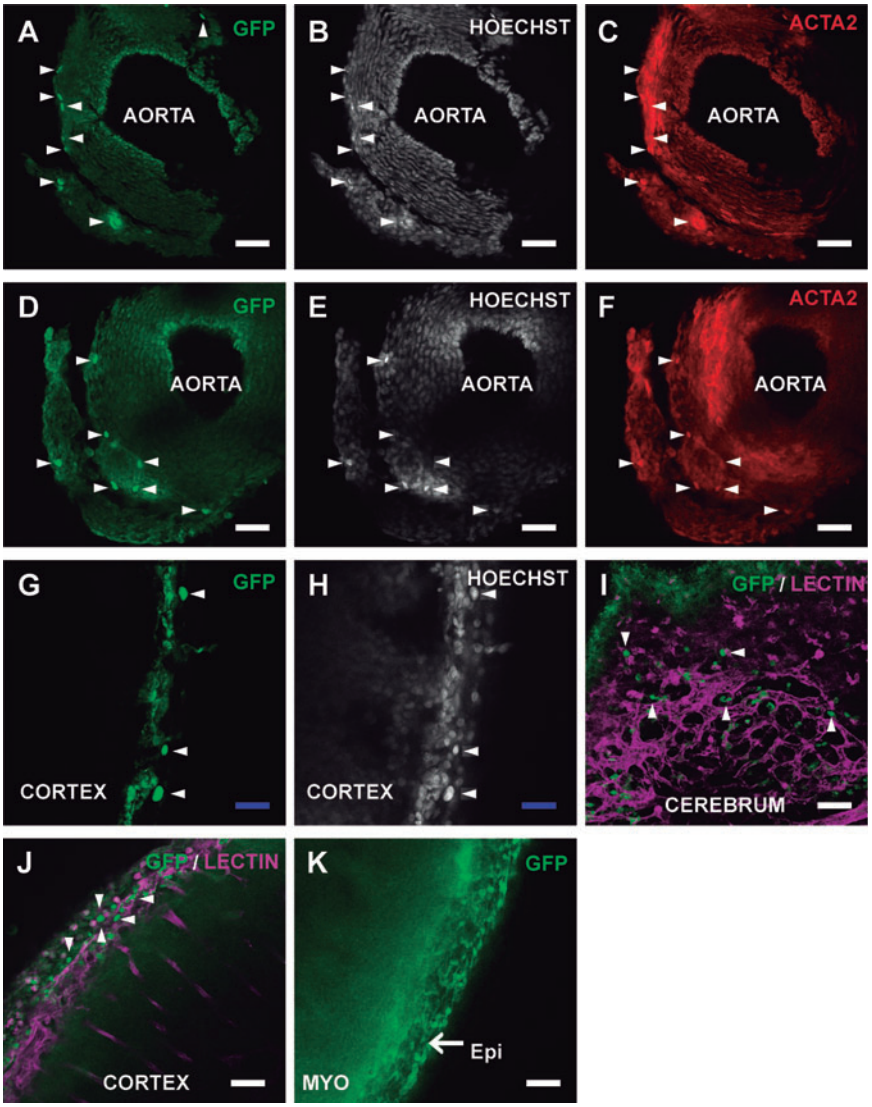

SUPPLEMENTARY FIG. S3. Transplanted NC cells localize specifically to NC locations in the chicken embryo. **(A–F)** Cross section of the chicken ascending aorta where fluorescent human NC cells were detected (*arrowheads*); **(B, E)** human cells identified by their bright and distinct Hoechst 33342 staining (*arrowheads*); **(C, F)** the human NC cells also expressed ACTA2 (*arrowheads*); **(G)** fluorescent human NC cells were seen in the cerebral cortex (parietal lobe) of chicken at HH stage 32 (*arrowheads*); **(H)** human cells identified by distinct Hoechst 33342 staining; **(I)** GFP^+ human NC cells identified in perivascular locations within a network of blood vessels (lectin stained) and within the cerebrum of chicken embryos; **(J)** GFP^+ human NC cells deposited in the cortex (frontal lobe) by blood vessels in the brain (identified by lectin); **(K)** myocardial and overlying epicardial (*arrow*) regions of the chicken embryo heart with no evidence of GFP^+ human NC cells. *White* scale bar: 100 µm, *blue* scale bar: 50 µm. Epi, epicardium; Myo, myocardium.
