# Supplementary material for: A Novel Human Pluripotent Stem Cell-Derived Neural Crest Model of Treacher Collins Syndrome Shows Defects in Cell Death and Migration
Source: Stem Cells Dev. 2019 Jan 10;28(2):81–100. doi: 10.1089/scd.2017.0234 (PMC6350417; doi:10.1089/scd.2017.0234)
Supplement: Supplemental data [file Supp_Fig4.pdf]

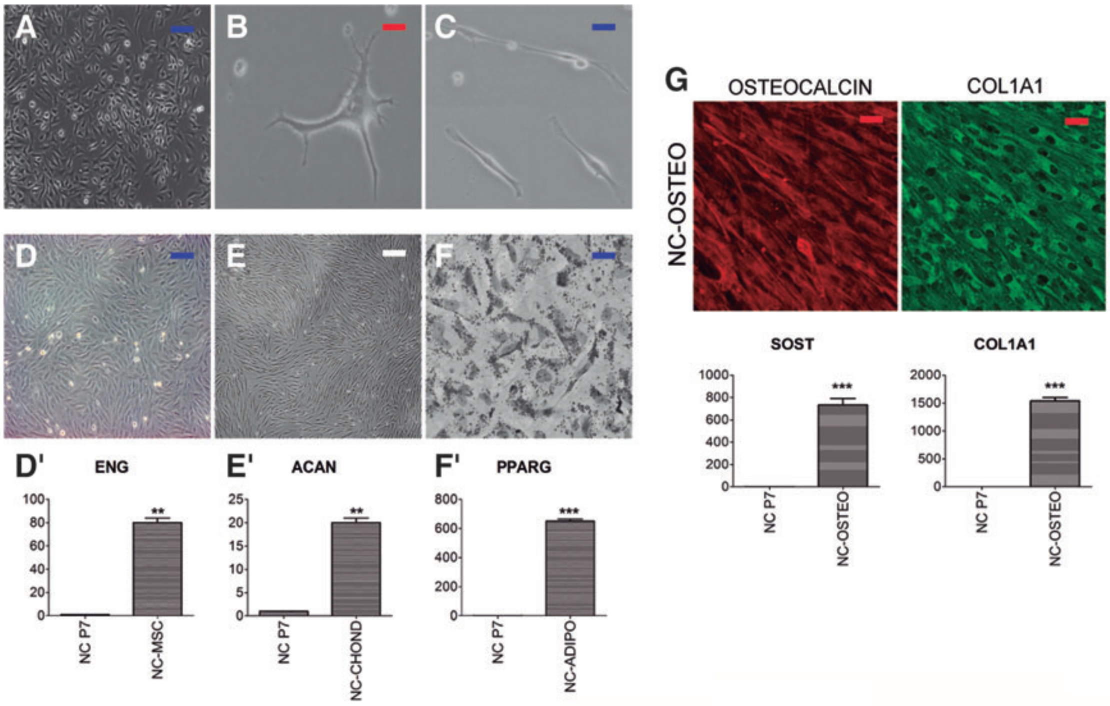

SUPPLEMENTARY FIG. S4. NC cells successfully differentiate into their derivatives. **(A)** Bright field image of representative HESC-derived NC cells. Scale bar 50 μm. **(B)** Bright field image of neurons derived from NC cells. Scale bar 20 μm. **(C)** Bright field image of representative melanocytes derived from NC cells. Scale bar 50 μm. **(D)** Bright field image of NC derived MSC. Scale bar 50 μm. **(D′)** qRT-PCR showing expression levels of the specific MSC marker Endoglin (*ENG*). The relative mRNA level was normalized to the housekeeping gene *PBGD*. Results are presented as mean ± SD of three independent experiments. $^{**}P < 0.01$, two-sided Student's *t*-test. **(E)** Bright field picture of representative NC P7 derived chondrocytes (NC-CHOND). Scale bar 100 μm. **(E′)** qRT-PCR showing expression levels of specific chondrocyte marker Aggrecan (*ACAN*). The relative mRNA level was normalized to the housekeeping gene *PBGD*. Results are presented as mean ± SD of three independent experiments. $^{**}P < 0.01$, two-sided Student's *t*-test. **(F)** Bright field picture of representative NC P7 derived adipocytes (NC-ADIPO). Scale bar 50 μm. **(F′)** qRT-PCR showing expression levels of specific adipocyte marker *PPARG*. The relative mRNA level was normalized to the housekeeping gene *PBGD*. Results are presented as mean ± SD of three independent experiments. $^{***}P < 0.001$, two-sided Student's *t*-test. **(G)** *Top panel*: Immunocytochemistry for Osteocalcin and COL1A1 in osteocytes derived from NC P7 (NC-OSTEO). Scale bar 20 μm. *Bottom panel*: qRT-PCR showing expression levels of the specific osteocyte genes Sclerostin (*SOST*) and Collagen1A1 (*COL1A1*). The relative mRNA level was normalized to the housekeeping gene *PBGD*. Results are presented as mean ± SD of three independent experiments. $^{***}P < 0.001$, two-sided Student's *t*-test. **(H)** qRT-PCR showing expression levels of the specific SMC genes *MYH11*, *MYOCD*, *TAGLN*, *SMTN-B*, and *ACTA2*, following SMC differentiation from NC-P7. The relative mRNA level was normalized to the housekeeping gene *PBGD*. Results are presented as mean ± SD of three independent experiments. $^{*}P < 0.05$; $^{**}P < 0.01$, two-sided Student's *t*-test. **(I)** qRT-PCR expression analysis of NC and SMC markers at different passages. *Top* graph: Expression analysis of specific NC genes (*P75, TFAP2A,* and *TWIST*) measured by qRT-PCR. The relative mRNA level was normalized to the housekeeping gene *PBGD*. The results are presented as mean ± SD of three independent experiments. $^{*}P < 0.05$; $^{**}P < 0.01$; $^{***}P < 0.001$. Two-sided Student's *t*-test. NC P1, NC differentiated from HPSC passage 1; NC P9, NC differentiated from HPSC passage 9; NC P7 + 2, NC differentiated from HPSC passage 7 when they were frozen. Those cells were thawed and passaged twice in FSB media before harvesting. *Bottom* graph: Expression level analysis of specific SMC genes (*ACTA2*, *CNN1*, *TAGLN*, *MYH11*) measured by qRT-PCR. The relative mRNA level was normalized to the housekeeping gene *PBGD*. The results are presented as mean ± SD of three independent experiments. $^{**}P < 0.01$, $^{***}P < 0.001$. Two-sided Student's *t*-test. NC P9, NC differentiated from HPSC passage 9; NC P9-SMC, SMC derived from NC P9; NC P7 + 2-SMC, SMC derived from NC cells that were frozen down at P7, thawed, and passaged twice in FSB media before commencing SMC differentiation. **(J)** Viability assay graph showing the percentage of viable NC P2 and P7 after thaw and culture for 3 h in FSB media. MSC, mesenchymal stem cells; SMC, smooth muscle cell.

(*continued*)

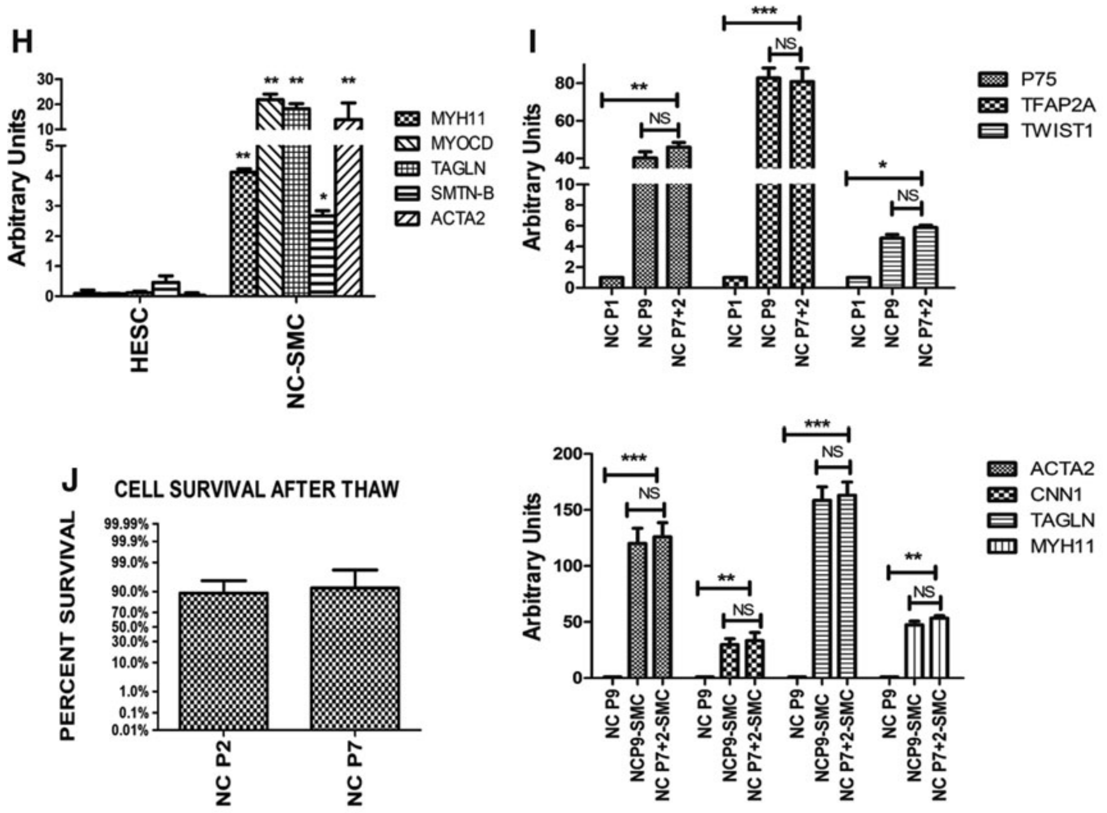

SUPPLEMENTARY FIG. S4. (Continued).
