# Supplementary material for: A Novel Human Pluripotent Stem Cell-Derived Neural Crest Model of Treacher Collins Syndrome Shows Defects in Cell Death and Migration
Source: Stem Cells Dev. 2019 Jan 10;28(2):81–100. doi: 10.1089/scd.2017.0234 (PMC6350417; doi:10.1089/scd.2017.0234)
Supplement: Supplemental data [file Supp_Fig5.pdf]

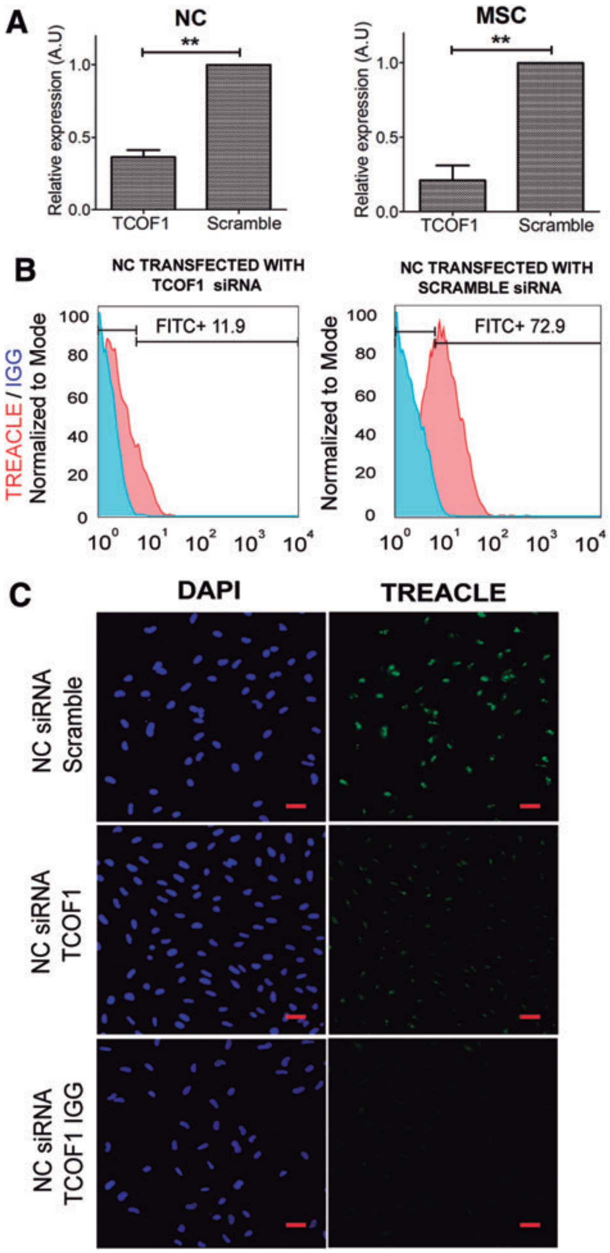

SUPPLEMENTARY FIG. S5. TCOF1 siRNA transient transfection in NC and MSC downregulates Treacle protein. **(A)** qRT-PCR showing the expression levels of TCOF1 in HESC-derived NC cells transfected with either TCOF1 siRNA or Scrambled siRNA (*left*). qRT-PCR showing the expression levels of TCOF1 in wild-type NC-derived MSC transfected with TCOF1 siRNA or scramble siRNA (*right*). The relative mRNA level was normalized to the housekeeping gene *PBGD*. The results are presented as mean ± SD of three independent experiments. $^{**}P < 0.01$, two-sided Student's *t*-test. **(B)** Histogram of the percentage of Treacle positive cells determined by flow cytometry analysis in HESC-derived NC cells transfected with TCOF1 siRNA or scrambled siRNA. *Red* histograms represent Treacle staining, and *blue* histograms represent IgG isotype controls. **(C)** Immunocytochemistry for Treacle protein (TCOF1) in HESC-derived NC transfected with either TCOF1 siRNA or Scramble siRNA. IgG is shown as a negative control. Scale bar 20 μm. siRNA, small interfering RNA.
