# Supplementary material for: A Novel Human Pluripotent Stem Cell-Derived Neural Crest Model of Treacher Collins Syndrome Shows Defects in Cell Death and Migration
Source: Stem Cells Dev. 2019 Jan 10;28(2):81–100. doi: 10.1089/scd.2017.0234 (PMC6350417; doi:10.1089/scd.2017.0234)
Supplement: Supplemental data [file Supp_Fig6.pdf]

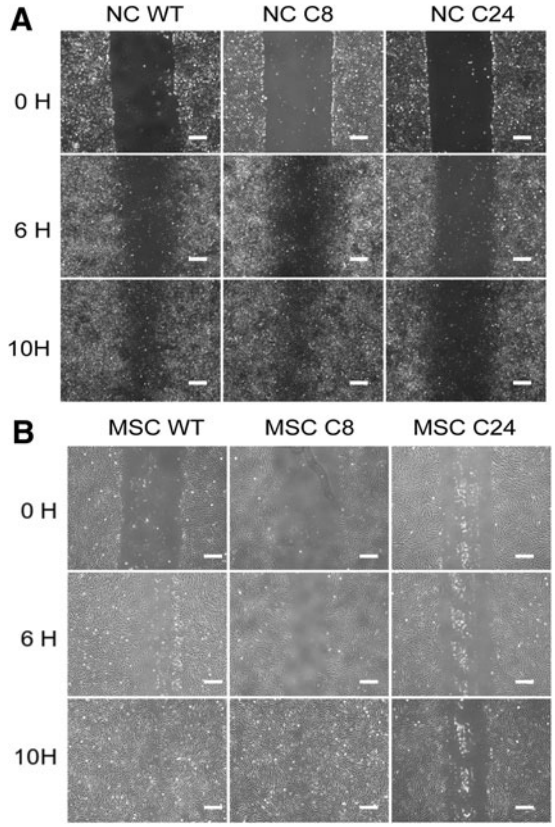

SUPPLEMENTARY FIG. S6. TCOF1$^{+/-}$ HIPSC-derived MSC show a defect in cell migration. **(A)** Representative images of scratch wound assay performed in TCOF1$^{+/+}$ NC derived from H9s (WT), HIPSC (C8), and NC derived from TCOF1$^{+/-}$ HIPSC (C24). Pictures were obtained at the indicated time points. Scale bar 100 μm. **(B)** Representative images of scratch assays carried out in MSC derived from NC TCOF1$^{+/+}$ (WT, C8) and NC TCOF1$^{+/-}$ (C24). Pictures were obtained at the indicated time points. Scale bar 100 μm.
